# Synthesis, biofilm formation inhibitory, and inflammation inhibitory activities of new coumarin derivatives

**DOI:** 10.1038/s41598-024-59072-w

**Published:** 2024-04-20

**Authors:** Ghada E. Ahmed, Zeinab A. Elshahid, Eslam Reda El-Sawy, Mohamed S. Abdel-Aziz, Anhar Abdel-Aziem

**Affiliations:** 1 High Canal Institute of Engineering & Technology, Suez, Egypt; 2 Chemistry of Natural and Microbial Products Department, Pharmaceutical and Drug Industries Research Institute, National Research Centre, Giza, Egypt; 3 Chemistry of Natural Compounds Department, National Research Centre, Dokki, Giza, 12622 Egypt; 4 Microbial Chemistry Department, Genetic Engineering and Biotechnology Institute, National Research Centre, Dokki, Giza, 12622 Egypt; 5 Chemistry Department, Faculty of Science (Girl’s Branch), Al-Azhar University, Cairo, 11754 Egypt

**Keywords:** Coumarin-6-sulfonyl chloride, 6-aminocoumarin, Heterocycles, Antimicrobial, Biofilm, Anti-inflammatory, Biofilms, Biochemistry, Drug discovery

## Abstract

Coumarins are heterocycles of great interest in the development of valuable active structures in chemistry and biological domains. The ability of coumarins to inhibit biofilm formation of Gram positive bacterium (*Staphylococcus aureus*), Gram negative bacterium (*Escherichia coli*) as well as the methicillin-resistant *S. aureus* (MRSA) has been previously described. In the present work, new hybrid coumarin-heterocycles have been synthesized via the reaction of coumarin-6-sulfonyl chloride and 6-aminocoumarin with different small heterocycle moieties. The biological efficacy of the new compounds was evaluated towards their ability to inhibit biofilm formation and their anti-inflammatory properties. The antimicrobial activities of the newly synthesized compounds were tested against Gram positive bacterium (*S. aureus* ATCC 6538), Gram negative bacterium (*E. coli* ATCC 25922), yeast (*Candida albicans* ATCC 10231) and the fungus (*Aspergillus niger* NRRL-A326). Compounds **4d**, **4e**, **4f**, **6a** and **9** showed significant MIC and MBC values against *S. aureus*, *E. coli*, *C. albicans*, and methicillin-resistant *S. aureus* (MRSA) with especial incidence on compound** 9** which surpasses all the other compounds giving MIC and MBC values of (4.88 and 9.76 µg/mL for *S. aureus*), (78.13 and 312.5 µg/mL for *E. coli*), (9.77 and 78.13 µg/mL for *C. albicans*), and (39.06 and 76.7 µg/mL for MRSA), respectively. With reference to the antibiofilm activity, compound **9** exhibited potent antibiofilm activity with IC_50_ of 60, 133.32, and 19.67 µg/mL against *S. aureus, E. coli,* and MRSA, (respectively) considering the reference drug (neomycin). Out of all studied compounds, the anti-inflammatory results indicated that compound **4d** effectively inhibited nitric oxide production in lipopolysaccharide-(LPS-) stimulated RAW264.7 macrophage cells, giving NO% inhibition of 70% compared to Sulindac (55.2%)

## Introduction

Coumarins (2*H*-1-benzopyran-2-ones) are an elite class of compounds present in various natural products, and they have wide applications including antiviral, antimicrobial, anti-inflammatory, and other bioactivities^1,2^. The incorporation of another heterocyclic moiety into coumarin enriches the properties of the parent structure and the resulting compounds may exhibit promising properties. Many examples of biologically active coumarins containing heterocycles-fused were cited in the literature including antimicrobial^3–5^, antiviral^6–8^, anticancer^9^, antioxidant, and anti-inflammatory activities^2,10–12^. On the other hand, the carbon–sulfur bond formation plays an important role in organic synthesis^13,14^. Remarkably, coumarin-coupled sulfonamide^15^, sulfonate^16^, sulfonohydrazide^17^ is an important structural motif that is a substantial template of an emerging class of therapeutic agents.

Biofilm inhibition is recognized as a novel drug target for the broad-spectrum anti-infective strategy to combat the infections caused by drug-resistant bacterial pathogens^18^. Some coumarins are approved to exhibits broad-spectrum antibiofilm activity against Gram-negative bacteria^19–23^. Coumarin derivatives has been reported to inhibit the biofilm formation of *Staphylococcus aureus*^24,25^, *Escherichia coli*^26^ and *Chromobacterium violaceum*^27^.

There is an increasing body of evidence suggests that anti-inflammatory drugs can exert some antimicrobial and anti-biofilm activities against clinically relevant pathogenic bacteria like *S. aureus*, *E. coli*, and MRSA^28^. In this respect, in vitro studies showed that NSAIDs such as diclofenac and ibuprofen have ensured that they have anti-biofilm activity in concentrations similar to those found in human pharmacokinetic studies. The mechanisms of anti-bacterial and anti-biofilm actions of NSAIDs differ according to the microbial species^28^. Based on the facts stated above, and in keeping with our ongoing search for new bioactive substances, a new series of coumarin-6-heterocyclics inspired by the adaptability of the coumarin moiety were synthesized and assessed for their antimicrobial activity against a variety of bacterial strains. The most potent substances were then tested for their ability to inhibit biofilm formation as well as evaluate their anti-inflammatory effects.

## Results and discussion

**Figure 1:**
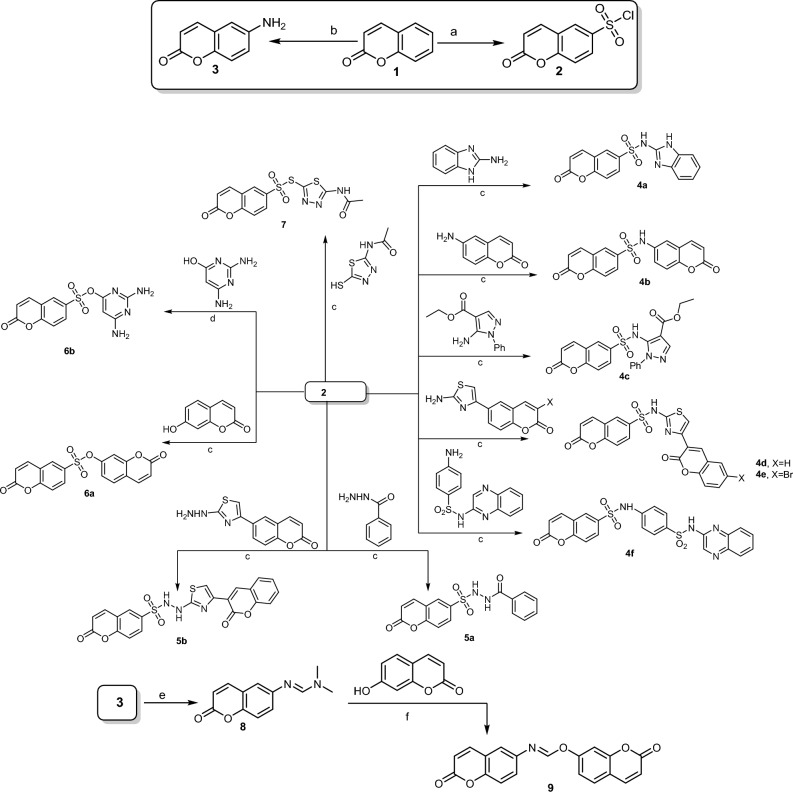
Synthetic pathway of new coumarin derivatives. Reagents and conditions: (**a**) HSO_3_Cl, 130−45 °C, 3 h; (**b**) i: HNO_3_, conc. H_2_SO_4_, 0–5 °C, 2 h, and then stirring at r.t., overnight; ii: stannous granules, TnCl_2_, conc. HCl, EtOH; 0–5 °C, 2 h, and then stirring at r.t., overnight; (**c**) EtOH, reflux, 5–30 min; (**d**) acetone, K_2_CO_3_, stirring, 70–80 °C; (**e**) dimethylformamide-dimethylacetal (DMF/DMA), dry xylene, reflux, 3 h; (**f**) EtOH, AcOH, reflux, 2 h.

In light of our aim to synthesize new active derivatives of coumarin-based sulfonamides, sulfonohydrazide, sulfonate, sulfothioate, and formimidate, we reported a simple and appropriate coupling reaction starting with coumarin-6-sulfonyl chloride (**2**) and 6-aminocoumarin (**3**) as shown in Fig. 1. The starting coumarin-6-sulfonyl chloride (**2**) was synthesized via the chlorosulfonation of coumarin (**1**) with chlorosulfonic acid under heating (130−45 °C)^29^. Whereas, 6-aminocoumarin was obtained via the reduction of 6-nitrocoumarin using stannous chloride in the presence of tin granule^30^.

The coupling reaction of compound (**2**) with various amino-heterocyclic namely, 2-aminobenzimidazole, 6-aminocoumarin, ethyl 5-amino-1-phenyl-1*H*-pyrazole-4-carboxylate, 3-(2-aminothiazol-4-yl)-2*H*-chromen-2-one^31^, 3-(2-aminothiazol-4-yl)-6-bromo-2*H*-chromen-2-one^32^, and 4-amino-*N*-(quinoxalin-2-yl)benzenesulfonamide in absolute ethanol under reflux led to the formation of the corresponding sulfonamides **4a-f** (Fig. 1). On the basis of elemental analyses and NMR spectral data, the chemical structures of the newly synthesized sulfonamides **4a-f** have been achieved. The ^1^H NMR spectra of **4a-f** showed the presence of additional aromatic protons besides the aromatic proton at ≈ δ 6.50 ppm that attributed to H-2 of the coumarin moiety. Their ^13^C NMR spectra also demonstrated the presence of the aliphatic peaks besides the aromatic carbons in their regions. For instance, the ^1^H NMR (DMSO-*d*_6_) spectrum of **4d** showed one singlet signal at δ 8.48 endorsing the presence of H-5 of thiazole moiety, besides a singlet signal at δ 7.51 ppm that back to NH proton. Also, the ^1^H NMR spectrum of **4d** showed two singlet signals at δ 6.45 and 6.47 ppm attributed to the presence of (two protons) at position-2 of two coumarin moieties, in addition to the aromatic protons located on their regions (Fig. s7). Its ^13^C NMR spectrum (DMSO-*d*_6_) demonstrated signals at δ 169.8 ppm (C-2, thiazole moiety), 160.4 (2 C=O), 158.7, 154.0, 153.0, 144.8, 144.5, 140.3, 133.2, 129.8, 129.4, 126.0, 125.6, 118.9, 118.4, 116.9, 116.5, 116.4, 109.1 ppm (C-Ar) (Fig. s8).

The building up of new coumarin-sulfonohydrazide derivatives was the next step of our work. The reaction of **2** with the prepared benzohydrazide^33^ and 6-bromo-3-(2-hydrazinylthiazol-4-yl)-2*H*-chromen-2-one^34^ in absolute ethanol under reflux gave the corresponding 2-oxo-2*H*-chromene-6-sulfonohydrazides **5a** and **5b** (Fig. 1).

The ^1^H NMR spectra were utilized to confirm the formation of the newly coumarin derivatives **5a,b**. For example, the ^1^H NMR (DMSO-*d*_6_) spectrum of **5a** displayed two singlet signals at δ 10.72, 10.22 ppm supporting the presence of two protons of NH, besides a doublet signal at δ 6.59 (*J* = 8.3 Hz) authorized the presence of H-2 of coumarin (Fig. s12).

On the other hand, compound **2** demonstrated its adaptability to react with more functional groups instead of the amino group via the reaction with the hydroxyl group of 7-hydroxycoumarin and 2,6-diaminopyrimidin-4-ol, besides thiol group of *N*-(5-mercapto-1,3,4-thiadiazol-2-yl)acetamide to afford coumarin-sulfonates (**6a**,**b**) and coumarin-sulfothioate (**7**), respectively (Fig. 1). The ^1^H NMR (DMSO-*d*_6_) spectrum of **6a** revealed one singlet signal at δ 8.74 which back to H-5 of the coumarin moiety, besides a doublet signal at δ 6.63 (*J* = 7.8 Hz) attributed to two CH protons of coumarin moieties at positions-2. The rest of the aromatic protons were located on their regions at δ 8.43 (d, *J* = 8.7 Hz, 2H), 8.17 (d, *J* = 9.3 Hz, 1H), 8.00 (d, *J* = 8.4 Hz, 1H), 7.75 (d, *J* = 8.4 Hz, 1H), 7.56 (d, *J* = 9.5 Hz, 2H), respectively (Fig. s13). Its ^13^C NMR (DMSO-*d*_6_) spectrum demonstrated signals at δ 159.4, 157.18 ppm supporting the presence of two C=O group, besides the remained aromatic carbon at δ 151.3, 151.2, 143.7, 137.1, 131.8, 130.2, 129.5, 128.3, 127.0, 123.3, 122.9, 118.4, 118.1 ppm (Fig. s14). On top of that, the ^1^H NMR (DMSO-*d*6) spectrum of compound **7** showed two singlet signals at δ 11.52 and 2.39 ppm endorsed the presence of (1H, NH) and (3H, CH_3_ of the acetyl protons), respectively, in addition to the aromatic protons which located on their regions at δ 8.53 (s, 1H), 7.82−7.66 (m, 1H), 7.58 (s, 1H), 6.54 (d, *J* = 8.6 Hz, 1H), 6.05 (s, 1H) (Fig. s16).

To increase the diversity of heterocyclic rings hybridized with coumarin, 6-aminocoumarin (**3**) was heated at reflux with *N,N*-dimethylformamide dimethyl acetal (DMF/DMA) to afford the corresponding enaminone (**8**) (Fig. 1). The acid catalytic reaction of compound **8** with 7-hydroxy coumarin in ethanol under reflux led to the formation of 2-oxo-2*H*-chromen-7-yl (*E*)-*N*-(2-oxo-2*H*-chromen-6-yl) formimidate (**9**) (Fig. 1). The NMR spectral data indicate the formation of compound **9**. The ^1^H NMR (CDCl_3_) spectrum of **9** showed one singlet signal at δ 8.71 ppm endorsed the presence of the anil proton (CH=N), besides two doublet signals at δ 6.74−6.69 (m, 1H), 6.40−6.34 (m, 1H) attributed of two (CH) of two coumarin moieties at position-2 (Fig. s18). Its ^13^C NMR (CDCl_3_) spectrum demonstrated signals at δ 176.7, 161.4 ppm of two C=O groups, in addition to one signal at δ 152.3 ppm of *C*H=N. Additionally, it revealed aromatic carbons at δ 147.9, 143.4, 138.4, 136.2, 127.8, 121.8, 119.4, 119.7, 117.9, 117.5, 116.8, 111.7, 110.2 ppm (Fig. s19).

## Biological evaluation

### Antimicrobial activity

Using the agar well diffusion assay, the newly synthesized coumarin derivatives, were estimated for their antimicrobial activity towards *S. aureus* (ATCC 6538),* E. coli* (ATCC 25933), *C. albicans* (ATCC 1023) besides *A. niger* (NRRL-A326)^35^. It has been found that the newly synthesized coumarin derivatives exhibited diverse activities in relation to the test microbe (Table 1, Fig. s1). Compounds **4f** and **9** had considerable antimicrobial activities against all test microbes with inhibition values of 16 and 16 mm against *S. aureus*, 15 and 9 mm against *E. coli*, 18 and 17 mm against *C. albicans,* and 15 and 19 mm against *A. niger*. It has been also found that compounds **4d**, **4e**, **6a**, and **7** had moderate activities against *S. aureus* with inhibition values of 13, 10, 14, and 12 mm (respectively), whereas the other compounds exhibited low or no activities against the same test microbe. For *E. coli*, compounds **4b**, **5b** and **6b** showed low activities with inhibition values of 7, 8 and 7 mm (respectively) and the other compounds showed negative results. On the other hand, compound **4d** had high activity with *C. albicans* (18 mm) but compounds **4e** and **6a** exhibited moderate activities (10 and 14 mm, respectively) whereas the other compounds showed low or no activities. For *A. niger*, compounds **4b**, **4c**, **4d**, **4e**, **5a**, **6a,** and **6b** had moderate activities (12, 13, 13, 12, 12, 14, and 12 mm, respectively), whereas the other compounds had low activities.

**Table 1:** In vitro antimicrobial activity of the newly synthesized coumarin derivatives against different test microbes using agar well diffusion method at concentration (250 μg/100μL).

Compounds no	Inhibition zone (ф mm)			
	*S. aureus* ATCC 6538	*E. coli* ATCC 25922	*C. albicans* ATCC 10231	*A. niger* NRRL A-326
**4a**	9	0	0	11
**4b**	6	7	9	12
**4c**	9	0	0	13
**4d**	13	0	18	13
**4e**	10	0	13	12
**4f**	16	15	18	15
**5a**	8	0	6	12
**5b**	0	8	7	8
**6a**	14	0	12	14
**6b**	8	7	9	12
**7**	12	0	0	7
**9**	16	9	17	19
neomycin	27	25	28	0
cycloheximide	0	0	0	22

Further works including minimum inhibition concentration (MIC) and minimum bactericidal concentration (MBC) had been done for the compounds that had comparatively high antimicrobial activates, **4d**, **4e**, **4f**, **6a** and **9** (Table 2, Fig. s2)^35,36^.

**Table 2:** The minimum inhibitory concentrations (MICs, μg/mL), and minimum bactericidal concentrations (MBCs, μg/mL) of **4d**, **4e**, **4f**, **6a** and **9.**

Compd. no	Pathogenic microorganisms							
	*S. aureus* ATCC 6538		*E. coli* ATCC 25922		*C. albicans* ATCC 10231		MRSA	
	MIC	MBC	MIC	MBC	MIC	MBC	MIC	MBC
**4d**	19.53	39.06	312.5	1250	156.25	312.5	316.7	624.4
**4e**	39.06	78.13	625	625	78.13	312.5	626.9	2500
**4f**	9.77	39.06	312.5	625	39.06	156.25	345.5	1400
**6a**	9.77	78.13	312.5	625	156.25	312.5	312.0	625
**9**	4.88	9.76	78.13	312.5	9.77	78.13	39.06	76.7
**Neomycin**	19.53	39.52	39.53	79.06	79.06	156.25	39.53	39.53

The data from Table 2 showed that, compound **9** exhibited the lowest MIC and MBC values for all test microbes with (4.88 and 9.76 µg/mL for *S. aureus*), (78.13 and 312.5 µg/mL for *E. coli*), (9.77 and 78.13 µg/mL for *C. albicans*), and (39.06 and 76.7 µg/mL for MRSA). Additionally, compounds **4f**, **6a**, **4d**, and **4e** showed remarkable promising MIC values 9.77, 9.77, 19.53, and 39.06 µg/mL (respectively) and MBC values 39.06, 78.13, 78.13, and 39.06 mL (respectively) against *S. aureus*. They also showed higher MIC and MBC values against the remained tested microbes. With respect to neomycin, compound **9** was about 4-fold more potent against *S. aureus*, followed by compound **4f** and **6a** (about 2-fold more potent). For *E. coli* compound **9** was less active than neomycin (2-fold lower). In case of *C. albicans*, both compounds **9** and **4f** were more potent than neomycin (4-fold and 2-fold, respectively, while compound **4e** was equipotent with neomycin. Out of the selected five compounds MIC study against MRSA, compound **9** was equipotent with neomycin.

Considering the structure–activity relationship after antimicrobial analysis, compounds **4f**, **6a**, and **9** showed the antimicrobial activity of the sulfonamide -containing coumarin and the presence of di-coumarin^37^. Interestingly, active compound **9** may confirm that the presence of two coumarin rings has a high antimicrobial effect against most of the microorganisms studied, consistent with what was previously reported^37^*.*

### Inhibition of biofilm formation

Microorganisms that can produce biofilms are known to be one of the major factors contributing to antibiotic resistance. Therefore, many experiments have been conducted to overcome these serious problems by searching for new drugs that can prevent biofilm formation^38^. *S. aureus* is one of the most frequent causes of biofilm-associated clinical infections. The increasing emergence of methicillin-resistant *S. aureus* (MRSA), antibiotic resistance, and biofilm-forming capacity contribute to *S. aureus* being the most commonly identified pathogen in both healthcare and community settings. Additionally, the ability to acquire novel antibiotic resistance mechanisms makes MRSA a major global health threat^24,39,40^.

Coumarin and its derivatives have attracted the attention of many microbiologists due to their antimicrobial effectiveness^41,42^. There are also further studies on the effectiveness of coumarin as an inhibitor of biofilm formation^20,43^. A recent study has proven that 3-hyrdroxy-coumarin, a marine bacterium-derived compound, showed antibiofilm formation^44^. So, the inhibition of biofilm formation was performed for the five most active compounds **4d**, **4e, 4f**, **6a**, and **9** using neomycin as a reference control compound. Table 3 and Fig. s3 explained the ability of the most active compounds with potent antibiofilm formation expressed as IC_50_ values. It was found that, compound **9** exhibited the best antibiofilm activities against *S. aureus*, *E. coli,* and MRSA with IC_50_ values of 60, 133.32, and 19.67 µg/mL, respectively in comparison to neomycin (IC_50_ = 19.67, 79.289, and 39.34 µg/mL, respectively). For the other compounds, it has been reported that compounds **4d** and **6a** had considerably acceptable results with *S. aureus* (IC_50_ of 185.51 and 355.52 µg/mL, respectively). The other compounds had appreciable IC_50_ values against the same test microbe. For *E. coli*, compounds **4d** and **4e** showed noticeable IC_50_ values 321.25 and 345.40 µg/mL (respectively). All the other compounds (**4d**, **4e**, **4f** and **6a**) when tested as antibiofilm formation by MRSA, compound **4e** and **6a** showed promising results (IC_50_: 85.02 and 40.73 µg/mL, respectively) in respect to neomycin. Out of the five selected compounds for inhibition of biofilm formation, compound **9** was the potent one and showing antibiofilm activity against MRSA (about twofold more potent) and about fivefold and threefold lower against *S. aureus*, *E. coli*, respectively.

**Table 3:** Inhibition of biofilm formation (IC_50_ µM) from *S. aureus*, *E. coli* and MRSA cultures treated with **4d, 4e, 4f, 6a**, and **9**.

Compd. no	Biofilm inhibition (IC_50_, µg\mL)		
	*S. aureus* ATCC 6538	*E. coli* ATCC 25922	MRSA
**4d**	185.51 ± 0.089	321.25 ± 0.24	99.54 ± 0.6
**4e**	584.49 ± 0.2	345.40 ± 0.22	85.02 ± 0.071
**4f**	624.90 ± 0.67	660.47 ± 0.24	386.12 ± 1.4
**6a**	355.52 ± 0.04	2644.16 ± 0.081	40.73 ± 1.6
**9**	60.00 ± 0.046	133.32 ± 0.23	19.67 ± 0.4
**Neomycin**	19.67 ± 0.300	79.289 ± 0.014	39.34 ± 0.011

Values are expressed as means ± SE; *n* = 3 for each group.

### Effect of compounds on nitric oxide levels in LPS-stimulated RAW 264.7 macrophages

**Figure 2:**
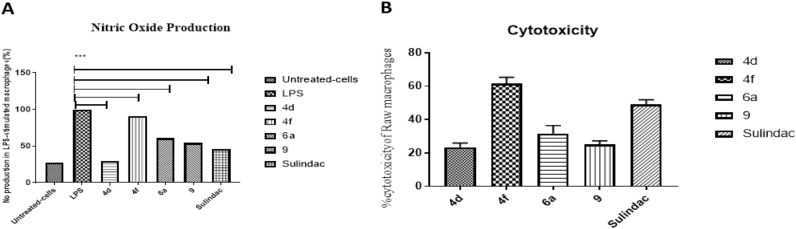
(**A**) Effects of the studied compounds on the production of nitric oxide (NO) in LPS-stimulated RAW264.7 macrophages. Cells were treated with the studied compounds at concentration 100 µg/mL plus LPS (1 μg/mL) or LPS alone for 24 h. Sulindac (NSAID) was used as a positive control. (**B**) Cytotoxic effect of the studied compounds and Sulindac on Raw-264.7 macrophages at concentrations (100 μg/mL). Values are expressed as the means ± SD (n = 3) *p* < 0.0001 (versus LPS alone, 2A).

Coumarins represent an important family of oxygen-containing heterocycles, widely distributed in nature^45,46^. Coumarin and its derivatives exhibited a broad range of biological and pharmacological activities^47^. A previous study indicated that imperatorin (a coumarin derivative) has an anti-inflammatory effect in lipopolysaccharide-stimulated mouse macrophages (RAW264.7) in an *in-vitro* model of edema, as it inhibits the protein expression of nitric oxide synthase (NOS) and a cyclooxygenase-2 (COX-2)^48^. The effect of the active compounds (**4d**, **4f**, **6a**, and **9**) on levels of Nitric Oxide (Fig. 2A) in LPS-stimulated RAW 264.7 cells was investigated according to the method of Elshahid et al.^49^. All the cells were treated with the studied compounds along with LPS or LPS alone for 24 h. To determine the level of NO production, the released of nitrite into the culture medium was measured using Griess reagent. As a result, LPS alone markedly induced NO production compared with that generated by the control. However, pretreatment with the studied compounds affected NO levels that significantly produced in LPS-stimulated RAW 264.7 cells as shown in Fig. 2A. Moreover, compounds (**4d**) induce marked inhibition on NO production by (70%) as compared to LPS (Table 4).

**Table 4:** The % inhibition of Nitric Oxide (NO) production in LPS-stimulated Raw Macrophages treated with the tested compounds **4d**, **4f**, **6a**, and **9**.

Sample code	Absorbance*	% Inhibition of NO
LPS (negative control)	0.317	0
4d	0.095	70
4f	0.287	9.5
6a	0.193	39.1
9	0.172	45.7
Sulindac (positive control)	0.142	55.2

*Results are represented as mean of 3 individual measurements.

In a parallel experiment, to examine the cytotoxicity of the studied compounds on RAW 264.7 cells, the cells were treated with each compound for 24 h in the presence or absence of LPS, and the cytotoxic potential was measured by the MTT assay^50^.

The results showed that compound (**4d**) was the least cytotoxic compound (≈ 20% cytotoxicity) indicating high cell viability. Meanwhile, compound (**4f**) and Sulindac (positive control) showed higher cytotoxic effect as indicated by the MTT reduction assay (Fig. 2B). These results clearly indicate that the anti-inflammatory activity of **4d** in LPS-stimulated RAW 264.7 macrophages was not due to direct cell death. Accumulating evidence indicates that NO is a critical mediators of inflammation^51,52^. NO plays a pivotal role in many body functions; however, its overproduction, particularly in macrophages, can lead to cytotoxicity, inflammation, and autoimmune disorders^51,52^. Our data are in agreement with several in-vitro studies performed with LPS-stimulated RAW264.7 cells, which showed that coumarin and its derivatives have shown a therapeutic effect against edema, eliminating proteins and fluid from injured tissue by activating mechanisms such as phagocytosis, enzyme release, and proteolysis^48,53,54^.

### Experimental part

#### Chemistry

All reagents and solvents were of commercial grade. Coumarin (Sigma-Aldrich Chemie GmeH, Taufkirchen, Germany). Melting points of the synthesized coumarins were measured on the digital melting point apparatus (Electro thermal 9100, Electro thermal Engineering Ltd., serial No. 8694, Rochford, United Kingdom) and are uncorrected. A Bruker Avance spectrometer (Bruker, Germany) was used to measure the ^1^H and ^13^C NMR spectra of new coumarins at 500 and 125 MHz, respectively. Elemental analyses were carried out on a Perkin–Elmer 2400 analyzer (USA) and were found within ± 0.4% of the theoretical values. TMS was used as the internal standard and hydrogen coupling patterns are described as (s) singlet, (d) doublet, (t) triplet, (q) quartet and (m) multiple. Chemical shifts were defined as parts per million (ppm) relative to the solvent peak.

***General Procedure for the preparation of coumarin sulfonamide derivatives* 4a-f**. An equal proportion of coumarin-6-sulfonyl chloride and applicable amino-compounds (10 mmol) in absolute ethanol (10 mL) was refluxed under stirring for 5–30 min. The precipitate formed on hot was collected by filtration and recrystallized from the proper solvent.

***N*-(1*H*-benzo[d]imidazol-2-yl)-2-oxo-2*H*-chromene-6-sulfonamide** **(4a)**. Recrystallized from ethanol\DMF as colorless crystals; MP. 295−7 °C; yield: 0.19 g, 55%; ^**1**^**H NMR** (500 MHz, DMSO-*d*_6_) δ 12.48 (s, 1H), 8.45 (s, 1H), 8.08 (t, *J* = 7.5 Hz, 1H), 7.95 (s, 1H), 7.77 (d, *J* = 8.3 Hz, 1H), 7.45−7.06 (m, 4H), 6.43 (t, *J* = 8.3 Hz, 1H), 6.14 (s, 1H). Analysis Calc. for C_16_H_11_N_3_O_4_S (341.34): C, 56.30; H, 3.25; N, 12.31; S, 9.39; Found: C, 56.41; H, 3.33; N, 12.44; S, 9.44.

**2-Oxo-*N*-(2-oxo-2*H*-chromen-6-yl)-2*H*-chromene-6-sulfonamide (4b).** Recrystallized from ethanol-DMF (5:1) as faint brown crystals; MP. 203−5 °C; yield: 0.30 g, 80%; ^**1**^**H NMR** (500 MHz, DMSO-* d*_6_) δ 10.56 (s, 1H), 8.12 (ddd, *J* = 12.1, 15.3, 7.3 Hz, 1H), 8.06−7.91 (m, 1H), 7.90−7.83 (m, 1H), 7.76 (dd, *J* = 11.0, 8.2 Hz, 1H), 7.59 (d, *J* = 10.9 Hz, 1H), 7.50 (dt, *J* = 10.2, 11.7 Hz, 1H), 7.41 (t, *J* = 9.7 Hz, 1H), 7.35−7.18 (m, 1H), 6.56 (td, *J* = 9.8, 6.1 Hz, 1H), 6.46 (ddd, *J* = 10.7, 9.4, 5.9 Hz, 1H). Analysis Calc. for C_18_H_11_NO_6_S (369.35): C, 58.54; H, 3.00; N, 3.79; S, 8.68; Found: C, 58.45; H, 2.98; N, 3.85; S, 8.73.

**Ethyl 5-((2-oxo-2*H*-chromene)-6-sulfonamido)-1-phenyl-1*H*-pyrazole-4-carboxylate (4c)**. Recrystallized from ethanol-DMF (5:1) as colorless crystals; MP. 220−2 °C; yield: 0.29 g, 65%; ^**1**^**H NMR** (500 MHz, DMSO-* d*_6_) δ 9.92 (dd, *J* = 11.7, 9.9 Hz, 1H), 9.61 (s, 1H), 8.28−8.16 (m, 1H), 8.09 (d, *J* = 12.1 Hz, 1H), 7.95 (dd, *J* = 2.4, 2.8 Hz, 1H), 7.85 (s, 1H), 7.82−7.71 (m, 1H), 7.68−7.28 (m, 4H), 6.55 (t, *J* = 8.0 Hz, 1H), 3.34 (q, *J* = 7.8.0 Hz, 2H), 1.01 (t, *J* = 6.7 Hz, 3H). Analysis Calc. for C_21_H_17_N_3_O_6_S (439.44): C, 57.40; H, 3.90; N, 9.56; S, 7.30; Found: C, 57.32; H, 3.98; N, 9.66; S, 7.22.

**2-Oxo-*N*-(2-(2-oxo-2*H*-chromen-3-yl)thiazol-4-yl)-2*H*-chromene-6-sulfonamide** **(4d)**. Recrystallized from ethanol-DMF (5:1) as green crystals; MP. 260−2 °C; yield: 0.41 g, 90%; ^**1**^**H NMR** (500 MHz, DMSO-* d*_6_) δ 8.48 (s, 1H), 8.09 (d, *J* = 9.5 Hz, 1H), 7.95 (s, 1H), 7.78 (d, *J* = 9.0 Hz, 1H), 7.73 (d, *J* = 7.6 Hz, 1H), 7.62 (t, *J* = 7.7 Hz, 1H), 7.51 (s, 1H), 7.42 (d, *J* = 8.3 Hz, 1H), 7.37 (t, *J* = 7.5 Hz, 1H), 7.32 (d, *J* = 8.5 Hz, 1H), 6.46 (2 s, 2H); ^**13**^**C NMR** (126 MHz, DMSO-* d*_6_) δ 169.8, 160.4, 158.7, 154.0, 153.0, 144.8, 144.5, 140.3, 133.2, 129.8, 129.4, 126.0, 125.6, 118.9, 118.4, 116.9, 116.5, 116.4, 109.1. Analysis Calc. for C_21_H_12_N_2_O_6_S_2_ (452.46): C, 55.75; H, 2.67; N, 6.19; S, 14.17; Found: C, 55.69; H, 2.77; N, 6.25; S, 14.22.

***N*-(2-(6-Bromo-2-oxo-2*H*-chromen-3-yl)thiazol-4-yl)-2-oxo-2*H*-chromene-6-sulfonamide (4e)**. Recrystallized from ethanol-DMF (5:1) as crystals; MP. 250−2 °C; yield: 0.43 g, 80%; ^**1**^**H NMR** (500 MHz, DMSO-* d*_6_) δ 8.39 (s, 1H), 8.12 (d, *J* = 9.6 Hz, 1H), 8.04 (d, *J* = 2.0 Hz, 2H), 7.95 (d, *J* = 1.6 Hz, 1H), 7.77 (dd, *J* = 8.5, 1.6 Hz, 1H), 7.70 (dd, *J* = 8.7, 2.1 Hz, 1H), 7.50 (s, 1H), 7.37 (t, *J* = 10.7 Hz, 1H), 7.31 (d, *J* = 8.5 Hz, 1H), 6.51 (d, *J* = 9.5 Hz, 1H). Analysis Calc. for C_21_H_11_BrN_2_O_6_S_2_ (531.35): C, 47.47; H, 2.09; Br, 15.04; N, 5.27; S, 12.07; Found: C, 47.51; H, 2.21; Br, 14.99; N, 5.33; S, 11.98.

**2-Oxo-N-(4-(N-(quinoxalin-2-yl)sulfamoyl)phenyl)-2H-chromene-6-sulfonamide** **(4f)** Recrystallized from ethanol-DMF (5:1) as colorless crystals; MP. 203−5 °C; yield: 0.28 g, 55%; ^**1**^**H NMR** (500 MHz, DMSO-* d*_6_) δ 12.84 (s, 2H), 7.92 (d, *J* = 8 Hz, 7H), 7.63 (s, 2H), 7.27 (s, 2H), 6.78 (d, *J* = 9.6 Hz, 3H). ^13^C NMR (126 MHz, DMSO-* d*_6_) δ 169.5, 167.1, 141.9, 135.5, 135.3, 131.9, 127.9, 127.0, 125.1, 124.1, 109.0; Analysis Calc. for C_23_H_16_N_4_O_6_S_2_ (508.05): C, 54.32; H, 3.17; N, 11.02; S, 12.61; Found: C, 54.35; H, 3.22; N, 12.43; S, 12.51.

**General Procedure for the preparation of coumarin sulfonohydrazide derivatives 5a and 5b.** These compounds were prepared as described for **4** from **2** (10 mmol) and benzohydrazide or 6-bromo-3-(2-hydrazinylthiazol-4-yl)-2*H*-chromen-2-one (10 mmol).

***N*'-benzoyl-2-oxo-2*H*-chromene-6-sulfonohydrazide (5a)**. Recrystallized from ethanol-DMF (5:1) as colorless crystals; MP. 235−7 °C; yield: 0.17 g, 50%; ^**1**^**H NMR** (500 MHz, DMSO-* d*_6_) δ 10.72 (s, 1H), 10.22 (s, 1H), 8.29−8.13 (m, 2H), 7.91 (dd, *J* = 9.4, 11.1 Hz, 2H), 7.65 (s, 2H), 7.49 (d, *J* = 8.8 Hz, 2H), 7.39 (s, 1H), 6.59 (d, *J* = 8.3 Hz, 1H). Analysis Calc. for C_16_H_12_N_2_O_5_S (344.34): C, 55.81; H, 3.51; N, 8.14; S, 9.31; Found: C, 55.78; H, 3.65; N, 8.22; S, 9.46.

**2-Oxo-*N*'-(2-(2-oxo-2*H*-chromen-3-yl)thiazol-4-yl)-2*H*-chromene-6-sulfonohydrazide (5b)**. Recrystallized from ethanol-DMF (5:1) as brown crystals; MP. 250−2 °C; yield: 0.42 g, 90%; ^**1**^**H NMR** (500 MHz, DMSO-* d*_6_) δ 9.68 (s, 1H), 9.59 (s, 1H), 8.31 (d, *J* = 8.5 Hz, 2H), 8.19−8.05 (m, 2H), 7.98−7.88 (m, 2H), 7.73 (t, *J* = 8.8 Hz, 2H), 7.54−7.20 (m, 2H), 6.47 (d, *J* = 9.5 Hz, 1H). Analysis Calc. for C_21_H_12_BrN_3_O_6_S_2_ (546.37): C, 46.16; H, 2.21; Br, 14.62; N, 7.69; S, 11.74. Found: C, 46.10; H, 2.30; Br, 14.72; N, 7.80; S, 11.82.

**Synthesis of 2-oxo-2*H*-chromen-7-yl 2-oxo-2*H*-chromene-6-sulfonate (6a)**. This compound was prepared as described for **4a** from **2** (10 mmol) and 7-hydroxy coumarin (10 mmol). The product (**6a**) recrystallized from ethanol-DMF (5:1) as yellow crystals; MP. 259−61 °C; yield: 0.22 g, 60%; ^**1**^**H NMR** (500 MHz, DMSO-* d*_6_) δ 8.74 (s, 1H), 8.43 (d, *J* = 8.7 Hz, 2H), 8.17 (d, *J* = 9.3 Hz, 1H), 8.00 (d, *J* = 8.4 Hz, 1H), 7.75 (d, *J* = 8.4 Hz, 1H), 7.56 (d, *J* = 9.5 Hz, 2H), 6.63 (d, *J* = 7.8 Hz, 2H); ^**13**^**C NMR** (126 MHz, DMSO-* d*_6_) δ 159.4, 157.18, . Analysis Calc. for C_18_H_10_O_7_S (370.33): C, 58.38; H, 2.72; S, 8.66; Found: C, 58.42; H, 2.65; S, 8.74.

**2,6-Diaminopyrimidin-4-yl 2-oxo-2H-chromene -6-sulfonate (6b)**. To a solution of 2,6-diaminopyrimidin-4-ol (10 mmol) in acetone containing (10 mmol) of K_2_CO_3_ was added compound 2 (10 mmol). The reaction mixture was stirring under heat (70–80 °C) for 4 h. The crude product was obtained upon filtration. Recrystallized from ethanol\DMF (5:1) as yellow crystals; MP. 255−7 °C; yield: 0.18 g, 55%; ^**1**^**H NMR** (500 MHz, DMSO-* d*_6_) δ 8.17 (d, *J* = 9.5 Hz, 1H), 7.78 (d, *J* = 8.1 Hz, 1H), 7.43 (s, 2H), 7.31 (t, *J* = 7.7 Hz, 1H), 6.48 (d, *J* = 9.5 Hz, 1H), 4.94 (s, 1H), 2.46 (s, 2H). Analysis Calc. for C_13_H_10_N_4_O_5_S (334.31): C, 46.71; H, 3.02; N, 16.76; S, 9.59; Found: C, 46.76; H, 3.12; N, 16.89; S, 9.60.

**Synthesis of *S*-(5-acetamido-1,3,4-thiadiazol-2-yl) 2-oxo-2*H*-chromene-6-sulfonothioate (7)**. This compound was prepared as described for **4a** from **2** (10 mmol) and *N*-(5-mercapto-1,3,4-thiadiazol-2-yl)acetamide (10 mmol). The product recrystallized from ethanol-DMF (5:1) as crystals; MP. 260−2 °C; yield: 0.27 g, 70%; ^1^H NMR (500 MHz, DMSO-*d*_*6*_) δ 11.52 (s, 1H), 8.53 (s, 1H), 7.82−7.66 (m, 1H), 7.58 (s, 1H), 6.54 (d, *J* = 8.6 Hz, 1H), 6.05 (s, 1H), 2.39 (s, 3H). Analysis Calc. for C_13_H_9_N_3_O_5_S_3_ (383.41): C, 40.72; H, 2.37; N, 10.96; S, 25.09; Found: C, 40.69; H, 2.40; N, 11.01; S, 24.98.

**Synthesis of (*E*)-*N,N*-Dimethyl-*N*'-(2-oxo-2*H*-chromen-6-yl)formimidamide (8)**. *N,N*-Dimethylformamide dimethyl acetal (10 mmol) was added to 6-aminocoumarin (10 mmol) in xylene (2 mL). The reaction mixture was heated at reflux for (3 h) then left to cool. The brown crystals formed after cooling were collected by scratch. Recrystallized from acetone as honey crystals; MP. 180 °C; yield: 0.13 g, 60%; ^**1**^**H NMR** (500 MHz, CDCl_3_) δ 7.72−7.50 (m, 2H), 7.29−7.08 (m, 2H), 7.03 (s, 1H), 6.43−6.30 (m, 1H), 3.08 (d, *J* = 8.8 Hz, 6H). Analysis Calc. for C_12_H_12_N_2_O_2_ (216.24): C, 66.65; H, 5.59; N, 12.96; Found: C, 66.56; H, 5.64; N, 13.00.

**Synthesis of 2-oxo-2*H*-chromen-7-yl (*E*)-*N*-(2-oxo-2*H*-chromen-6-yl) formimidate** **(9).** Compound 8 (10 mmol) and 7-hydroxy coumarin (10 mmol) in absolute ethanol (10 mL) containing (0.5 mL) of glacial acetic acid was heated under reflux for 2 h. The crude product was collected and recrystallized from acetone as brown crystals; MP. 108−10 °C; yield: 0.2 g, 60%; ^**1**^**H NMR** (500 MHz, CDCl_3_) δ 8.71 (s, 1H), 8.17 (d, *J* = 9.3 Hz, 1H), 7.60−7.54 (m, 1H), 7.44 (tdd, *J* = 9.2, 5.8, 3.0 Hz, 1H), 7.36−7.30 (m, 1H), 7.27−7.24 (m, 1H), 7.20−7.16 (m, 1H), 7.13 (d, *J* = 8.8 Hz, 1H), 6.90−6.83 (m, 1H), 6.74−6.69 (m, 1H), 6.40−6.34 (m, 1H); ^**13**^**C NMR** (126 MHz, CDCl_3_) δ 176.7, 161.4, 152.3, 147.9, 143.4, 138.4, 136.2, 127.8, 121.8, 119.4, 119.7, 117.9, 117.5, 116.8, 111.7, 110.2; Analysis Calc. for C_19_H_11_NO_5_ (333.30): C, 68.47; H, 3.33; N, 4.20; Found: C, 68.32; H, 3.21; N, 4.44.

### Biological assays

#### Antimicrobial assay

The antimicrobial activity of the synthesized compounds were assessed against *Staphylococcus aureus* ATCC 6538-P as Gram positive bacterium, *Escherichia coli* ATCC 25933 as Gram negative bacterium, *Candida albicans* ATCC 10231 as yeast as well as the filamentous fungal test microbe *Aspergillus niger* NRRL-A326 by the agar well diffusion method^35^. Bacterial and yeast test microbes were inoculated on nutrient agar medium plates seeded with 0.1 mL of 10^5^–10^6^ cells/mL whereas the fungal test strain was cultivated on plates having potato dextrose agar medium that seeded by 0.1 mL (10^6^ cells/mL) of the fungal inoculum. 5 mg of each sample was dissolved in 2 mL of DMSO. 100 µl from each sample were distributed in holes developed in each inoculated plate. Then plates were kept at 4 °C for more than 2 h to allow extreme dispersion. The plates were then kept at 37 °C overnight for bacteria and yeast and kept at 30 °C for 2 days for the fungus in vertical location to permit maximum microbial growth. Neomycin was used as reference drug for Gram-positive and Gram-negative bacteria as well as yeast. Cyclohexamide was used as reference drug for fungi (*A. niger*). The clear zone diameters expressed in millimeter (mm) were used to differentiate the antimicrobial activity of tested compounds. The experiment was carried out twice and their mean were considered.

#### Evaluation of minimum inhibitory concentration (MIC) and Minimum bactericidal Methicillin Resistant * S. aureus* concentration (MBC)

MIC was performed using *S. aureus* ATCC 6538, Gram-positive bacterium, and *E. coli* ATCC 25922, Gram-negative bacterium, *Candida albicans* ATCC 10231 as yeast, and Methicillin Resistant *S. aureus* (MRSA) as tested microbes that are grown on a Mueller Hinton medium. Test microbes were cultivated in 100 mL bottles with each test at 35 °C for 24 h. Cells were obtained by centrifugation (4000 rpm) under a sterile condition at 4 °C for 15 min. The cells were washed using sterile saline until the supernatant was clear. Cells with an optical density of 0.5 to 1 (at 550 nm) giving an actual number of colony-forming units of 5 × 10^6^ cfu/mL were obtained. Resazurin solution was prepared by dissolving 270 mg tablet in 40 mL of sterile distilled water. Then, 96-well sterile microplates were prepared. Then, 50μL of test material in DMSO was pipetted into the first row of the plate. To all other wells, 50μL of broth medium was added. Two-fold serial dilutions were performed. Then, 10μL of resazurin indicator solution was added, 10μL of bacterial suspension was added to each well. The plates were prepared in duplicate and placed in an incubator set at 37 °C for 18–24 h. Any colour changes from purple to pink or colourless were recorded as positive. The lowest concentration at which colour change occurred was taken as the MIC value. MBC has been performed by streaking of the two concentrations higher than MIC and the plates exhibiting no growth were considered as MBC^35,36^. Neomycine has been used as positive control^55^.

#### Inhibition of biofilm formation (crystal violet method)

Bacterial strains were incubated in test tubes with TSB (5 mL) containing 2% w/v glucose at 37 °C for 24 h. After that, the bacterial suspensions were diluted to achieve turbidity equivalent to a 0.5 McFarland standard. The diluted suspension (2.5μL) was added to each well of a single cell culture polystyrene sterile, flat-bottom 96-well plate filled with TSB (200μL) with 2% w/v glucose. Sub-MIC concentration values of compounds **4d, 4e, 4f, 6a**, and **9** were directly added to the wells to reach concentrations ranging from 100 to 0.1 μM to assess BIC_50_ values that are, the concentration at which the percentage of inhibition of biofilm formation is equal to 50%. Plates were incubated at 37 °C for 24 h. After biofilm growth, the content of each well was removed, wells were washed twice with sterile NaCl 0.9% and stained with 200μL of 0.1% w/v crystal violet solution for 15 min at 37 °C. The excess solution was removed, and the plate was washed twice, using tap water. A volume of 200 μL of ethanol was added to each stained well to solubilize the dye^35,38^. Neomycine has been used as positive control^55^. Optical density (O.D.) was read at 600 nm using a microplate reader (GloMax®-Multi Detection System, Milan, Italy). The experiments were run at least in triplicates, and three independent experiments were performed. The percentage of inhibition was calculated through the formula:

1 $$ \% {\text{ Inhibition}} = \left( {{\text{OD}}\;{\text{growth}} - {\text{OD}}\;{\text{sample}}/{\text{OD}}\;{\text{growth}}\;{\text{control}}} \right) \times {1}00 $$

### Anti‑inflammatory assay

#### Cell culture (seeding and treatment)

The RAW 264.7 macrophage cell line were supplied from ATCC (American type culture collection).The cells were sub-cultured in Roswell Park Memorial Institute's RPMI 1640 medium^49^.

#### Procedure

The following procedures were all completed in a biosafety level II Laminar flow cabinet in a clean environment. RAW 264.7 cells were suspended at concentration of 1 × 10^**5**^ cells per well (in 96 well plates). The cells were then incubated with the test compounds, LPS (lipopolysaccharide, negative control) and Sulindac (positive control drug) according to the method^49^*.* After 24 h, the supernatant was gently transferred to new 96-well plates for measuring nitric oxide (NO) while cells were used for cell viability testing using the MTT method. The percentage change in viability was calculated according to the below formula

$$ \left( {\left( {{\text{Reading}}\;{\text{of}}\;{\text{extract}}/{\text{Reading}}\;{\text{of}}\;{\text{negative}}\;{\text{control}}} \right) - {1}} \right) \times {1}00 $$

#### Nitric oxide assay

The generation of nitric oxide (NO) was measured in the supernatants of cultivated RAW 264.7 cells. With slight modification, the Nitric Oxide (NO) measurement was carried out as described by Eid et al.^50^ using the Griess reagent. In detail, 50 μl of cell culture media were added to 50 μl of Griess reagent then incubated at room temperature for 15 min before being measured at 540 nm^50^. A sodium nitrite standard curve was used to calculate the amount of nitrite, as shown in equation:

$$ {\text{Nitric}}\;{\text{Oxide}}\;{\text{inhibition}}\left( \% \right) = \frac{{\left( {{\text{control}} - {\text{Test}}} \right)}}{{ {\text{control}}}} \times {1}00 $$

#### Statistical analysis

All statistical analysis and IC_50_ values were calculated using the concentration–response curve fit to the non-linear regression model and One-way ANOVA with Dunnet’s posttest was performed using GraphPad Prism® v7.0 (GraphPad Software Inc., San Diego, CA, USA).

## Conclusion

Coumarins are considered as brilliant groups of compounds existed in nature with diverse chemical skeleton and biological activities. This study focused on the synthesis of new coumarin-conjugated sulfonamides, sulfonohydrazide, sulfonate, sulfothioate, and formimidate. The new compounds were assessed as antimicrobial, antibiofilm, and anti-inflammatory activities. 2-oxo-2*H*-chromen-7-yl (*E*)-*N*-(2-oxo-2*H*-chromen-6-yl) formimidate (**9**) exhibited brilliant antimicrobial activity, as well as antibiofilm activity. On the other side, 2-oxo-*N*-(2-(2-oxo-2*H*-chromen-3-yl)thiazol-4-yl)-2*H*-chromene-6-sulfonamide (**4f**) effectively inhibited nitric oxide production in lipopolysaccharide- (LPS-) stimulated RAW264.7 macrophage cells and could be considered as anti-inflammatory agent. Noteworthy, it is not a requirement, that a compound that has a strong antimicrobial effect may play an anti-inflammatory role. This may be due to the role of the active groups in the two compounds.

### Supplementary Information

Supplementary Information.

## Data Availability

All data generated or analyzed during this study are included in this published article [and its supplementary information files].
