# Supplementary material for: Synthesis, biofilm formation inhibitory, and inflammation inhibitory activities of new coumarin derivatives
Source: Sci Rep. 2024 Apr 20;14:9106. doi: 10.1038/s41598-024-59072-w (PMC11032357; doi:10.1038/s41598-024-59072-w)
Supplement: Supplementary file 1 — Supplementary Information. [file 41598_2024_59072_MOESM1_ESM.docx]

**Synthesis, Biofilm Formation Inhibitory, and Inflammation Inhibitory Activities of New Coumarin Derivatives**


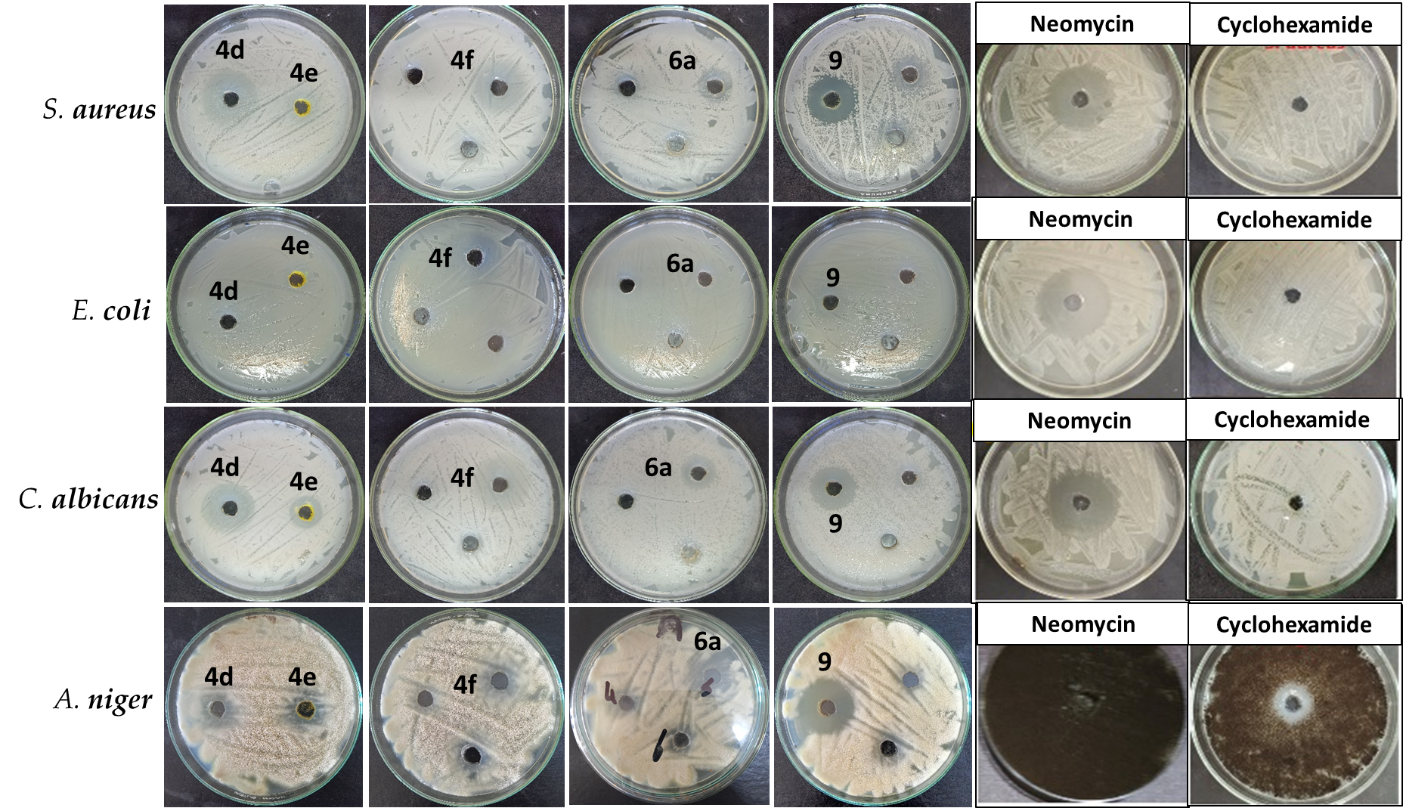


**Figure s1**. The inhibition zone (фmm) of the most active compounds, **4d**, **4e**, **4f**, **6a**, and **9**, besides the reference drugs, neomycin (standard antibacterial) and cyclohexamide (standard antifungal) against various pathogenic microorganisms


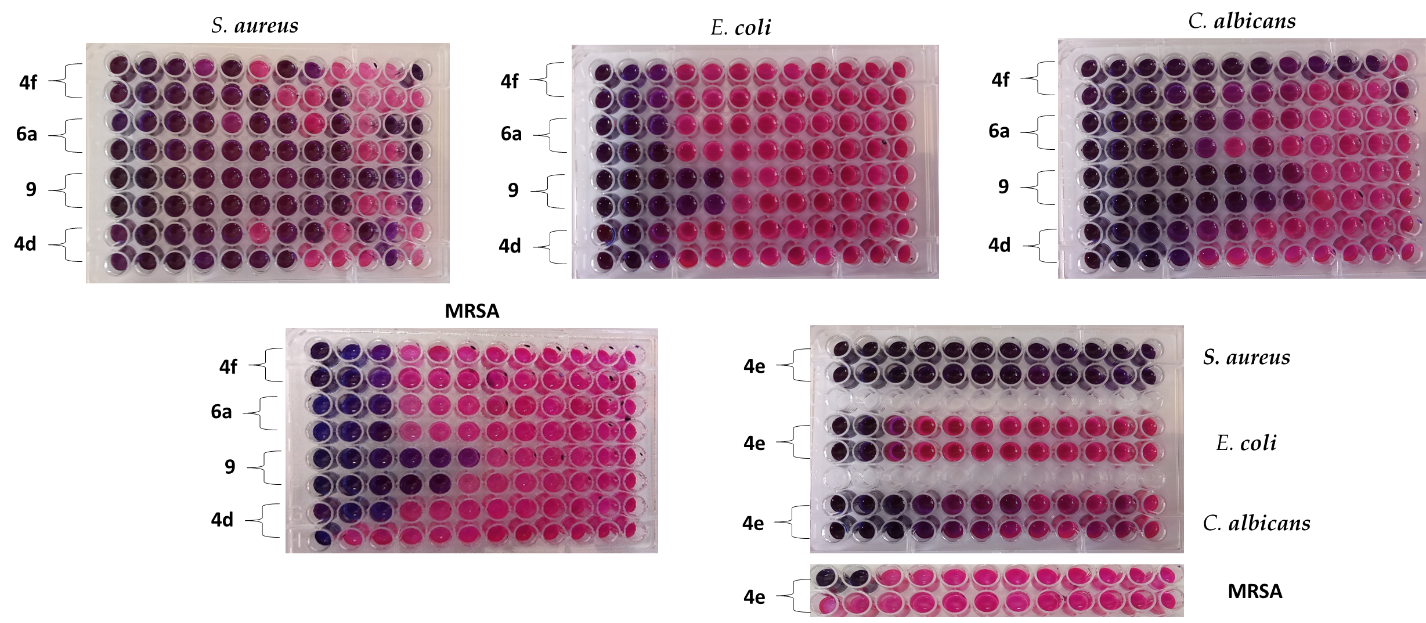


**Figure s2.** The inhibitory concentrations (MICs) of compounds **4d**, **4e**, **4f**, **6a**, and **9** against *S. aureus*, *E. coli*, *C. albicans*, and MRSA

**
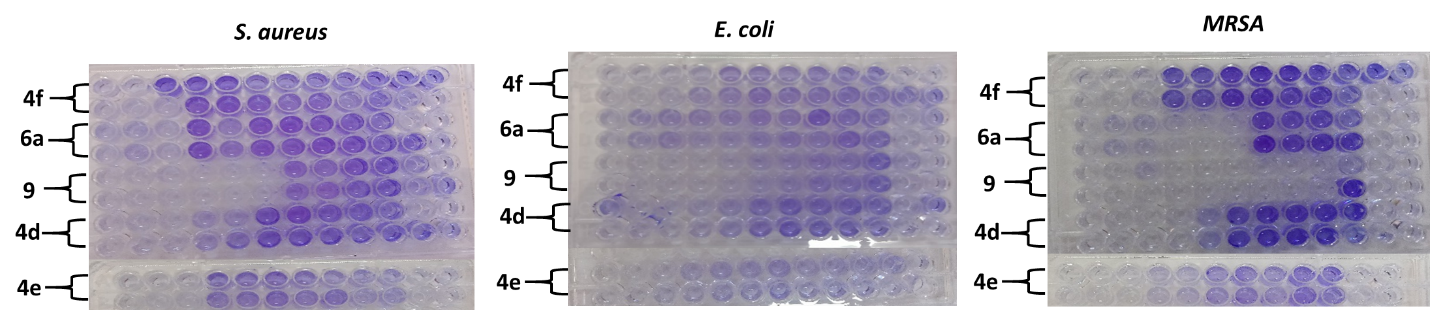
**

**Figure s3.** Inhibition of bacterial biofilm formation using **4d**, **4e**, **4f**, **6a**, and **9** against *S. aureus*, *E. coli*, and MRSA. Values are expressed as means ± SE; *n* = 3 for each group.


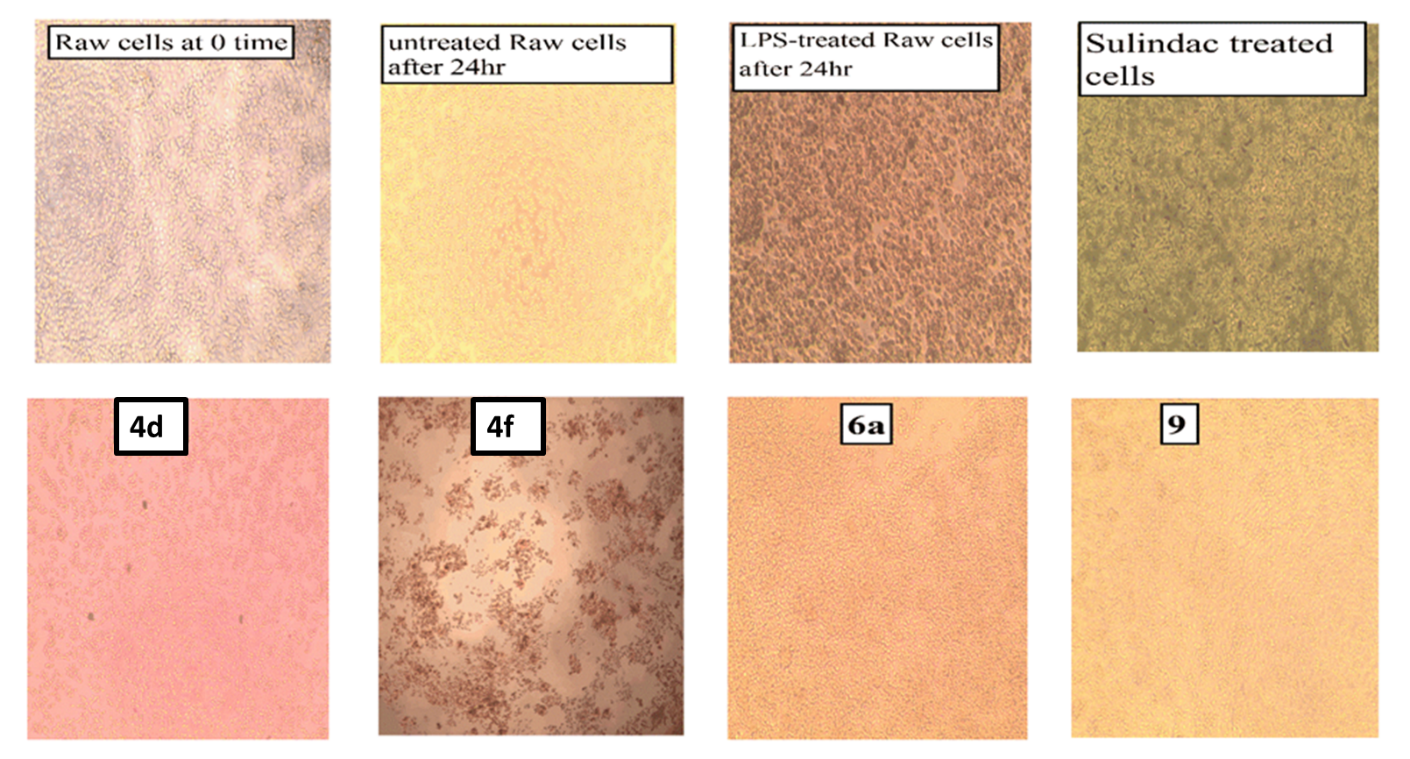


**Figure s4.** Viability of RAW 264.7 macrophage cells exposed to different compounds. Images were acquired at 100ppm after 24hr using phase contrast microscope. LPS was used as negative control while, Sulindac is the positive control. Imaging in 96-well microplates were carried out manually on OlympusCKX41- inverted phase-contrast microscope, cellsens software.


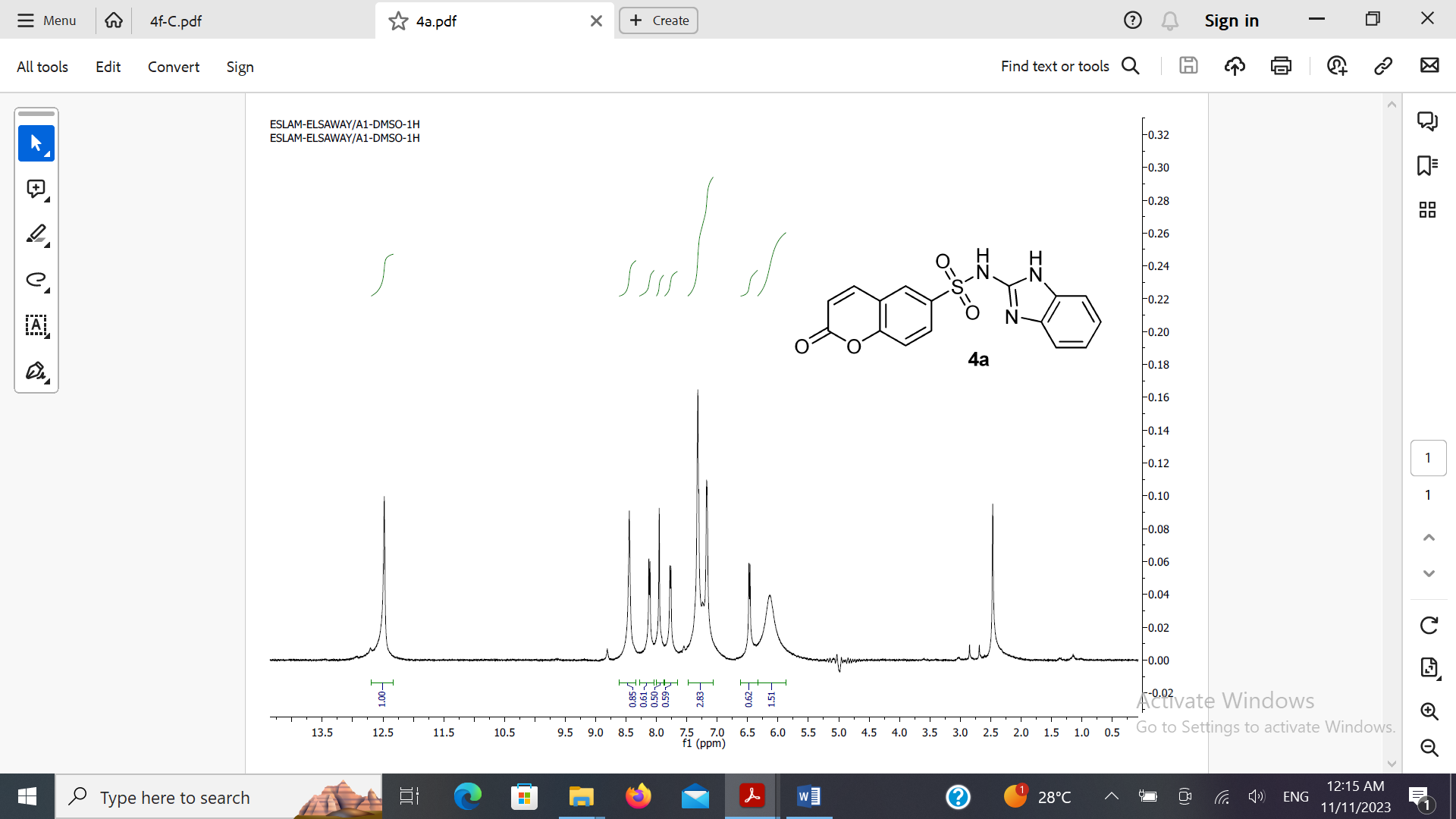


Figure s5. The ^1^H NMR (DMSO-d_6_) spectrum of compound **4a**


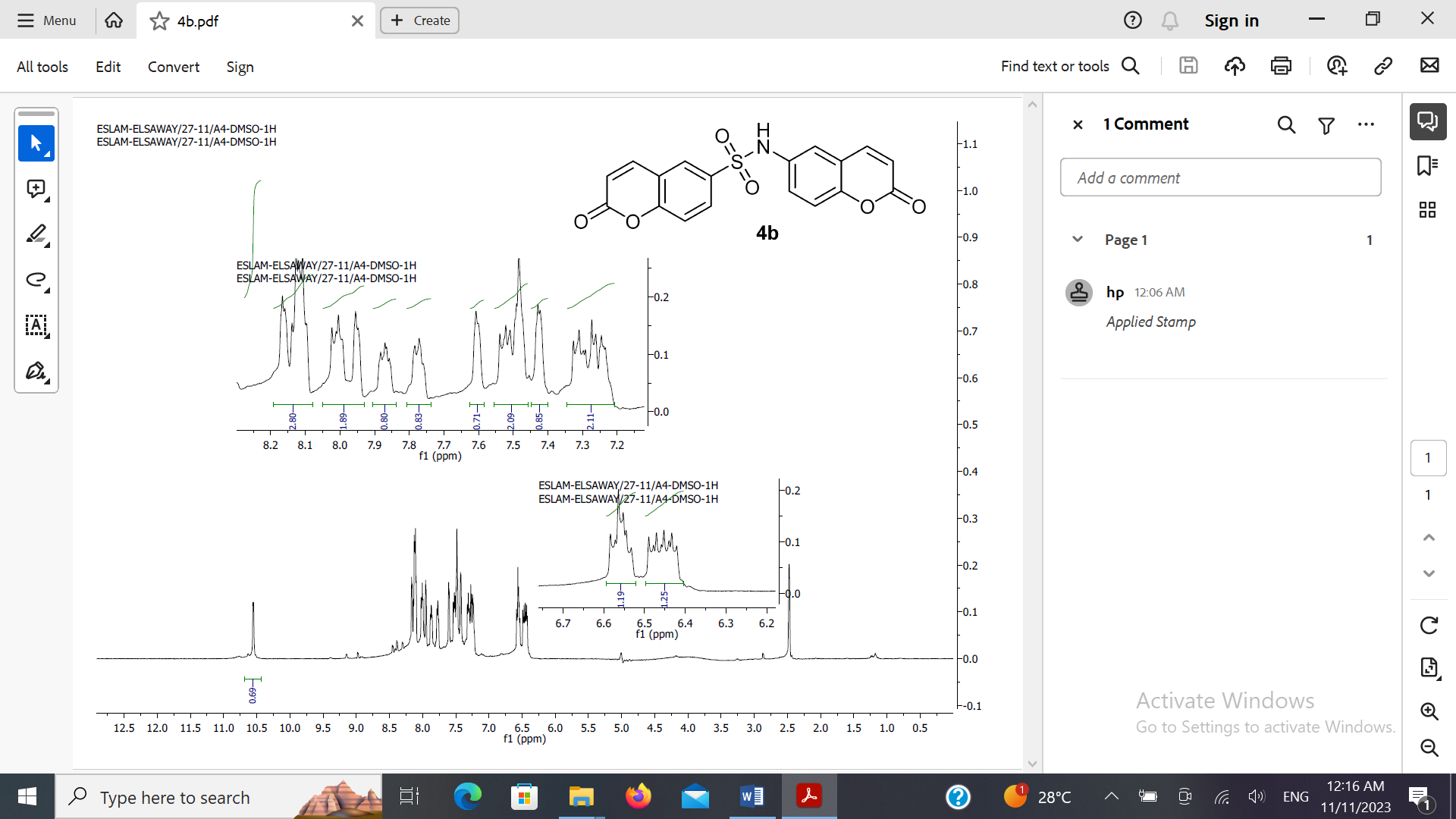


Figure s6. The ^1^H NMR (DMSO-d_6_) spectrum of compound **4b**


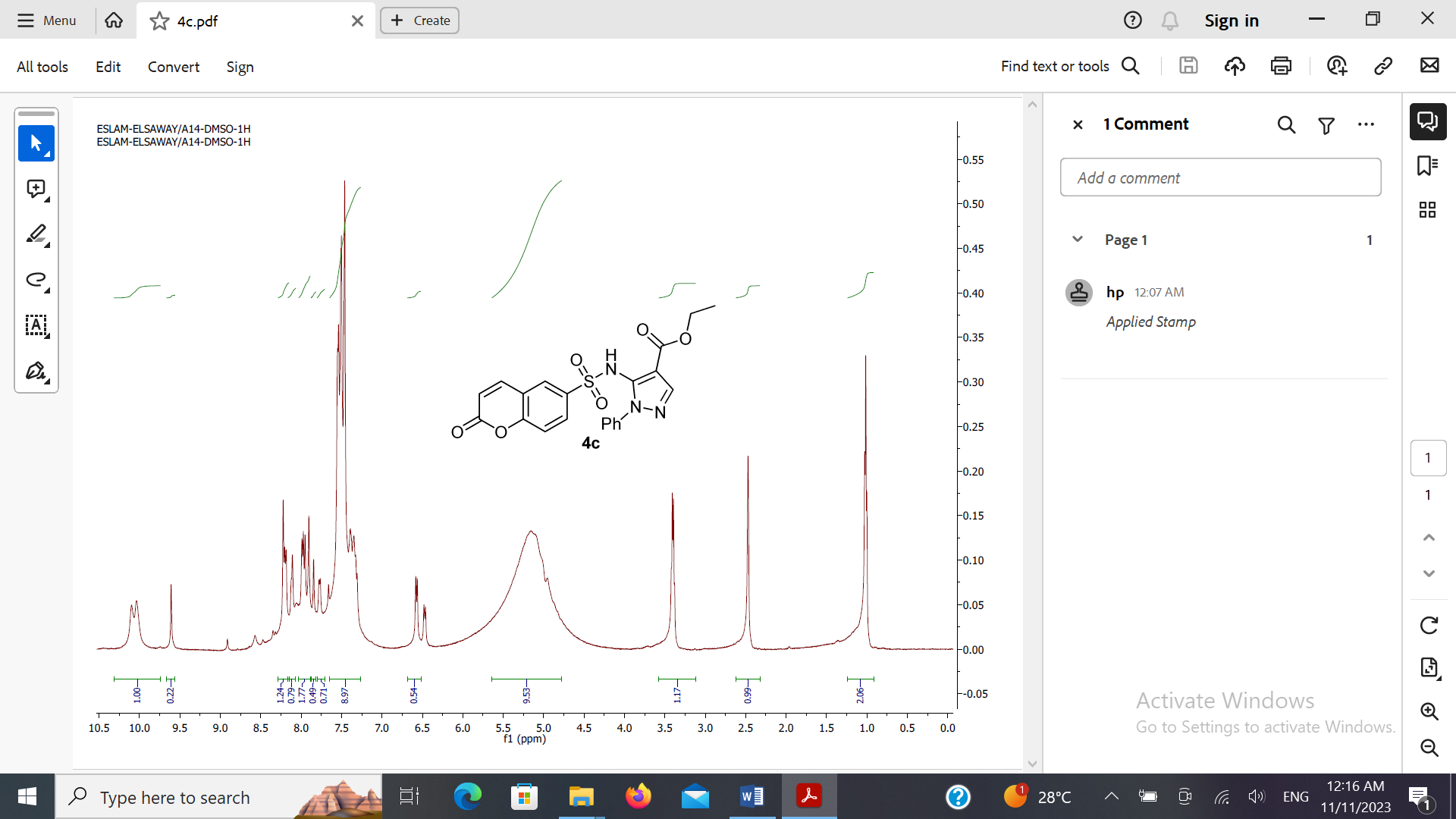


Figure s7. The ^1^H NMR (DMSO-d_6_) spectrum of compound **4c**


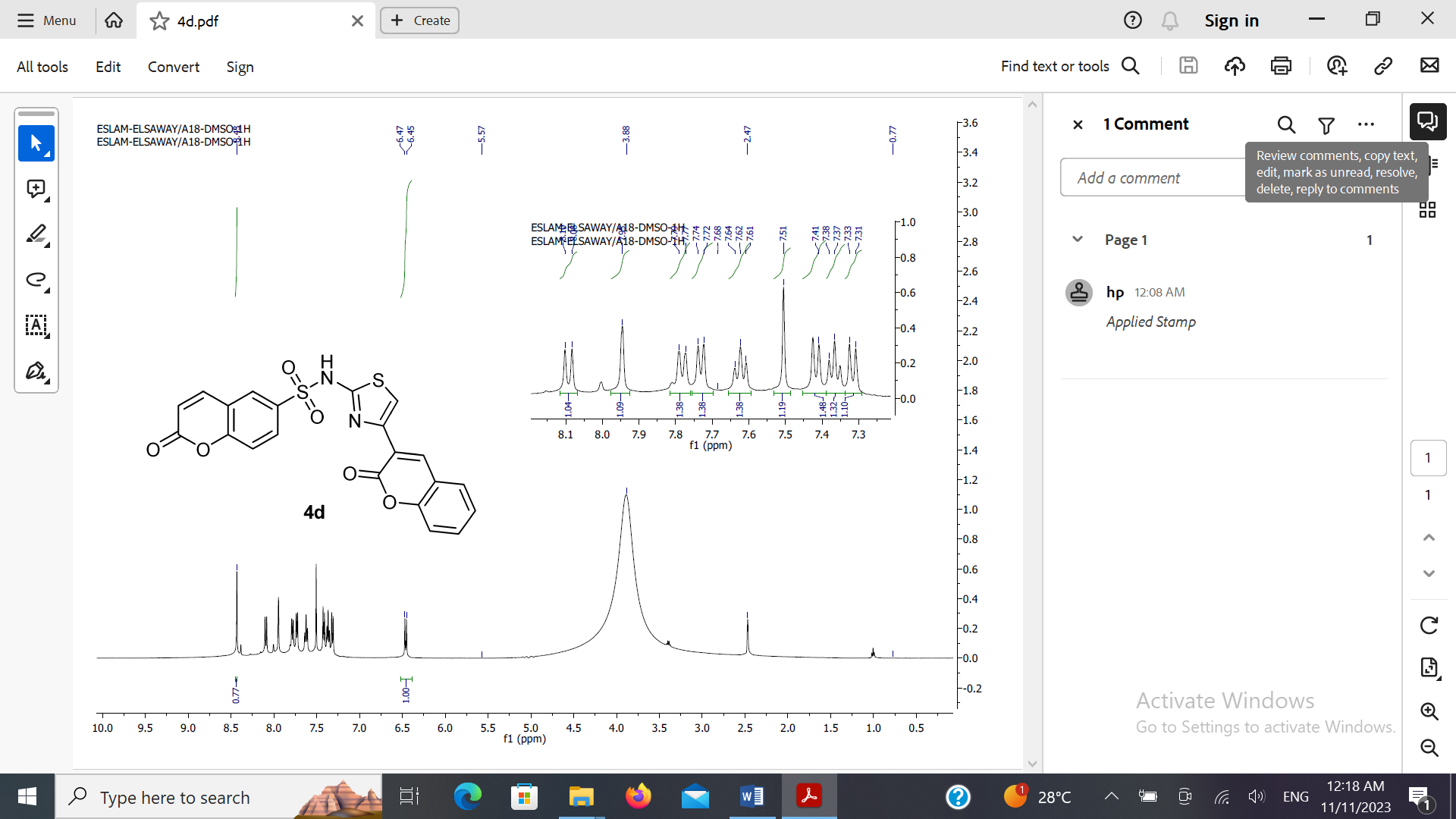


Figure s8. The ^1^H NMR (DMSO-d_6_) spectrum of compound **4d**


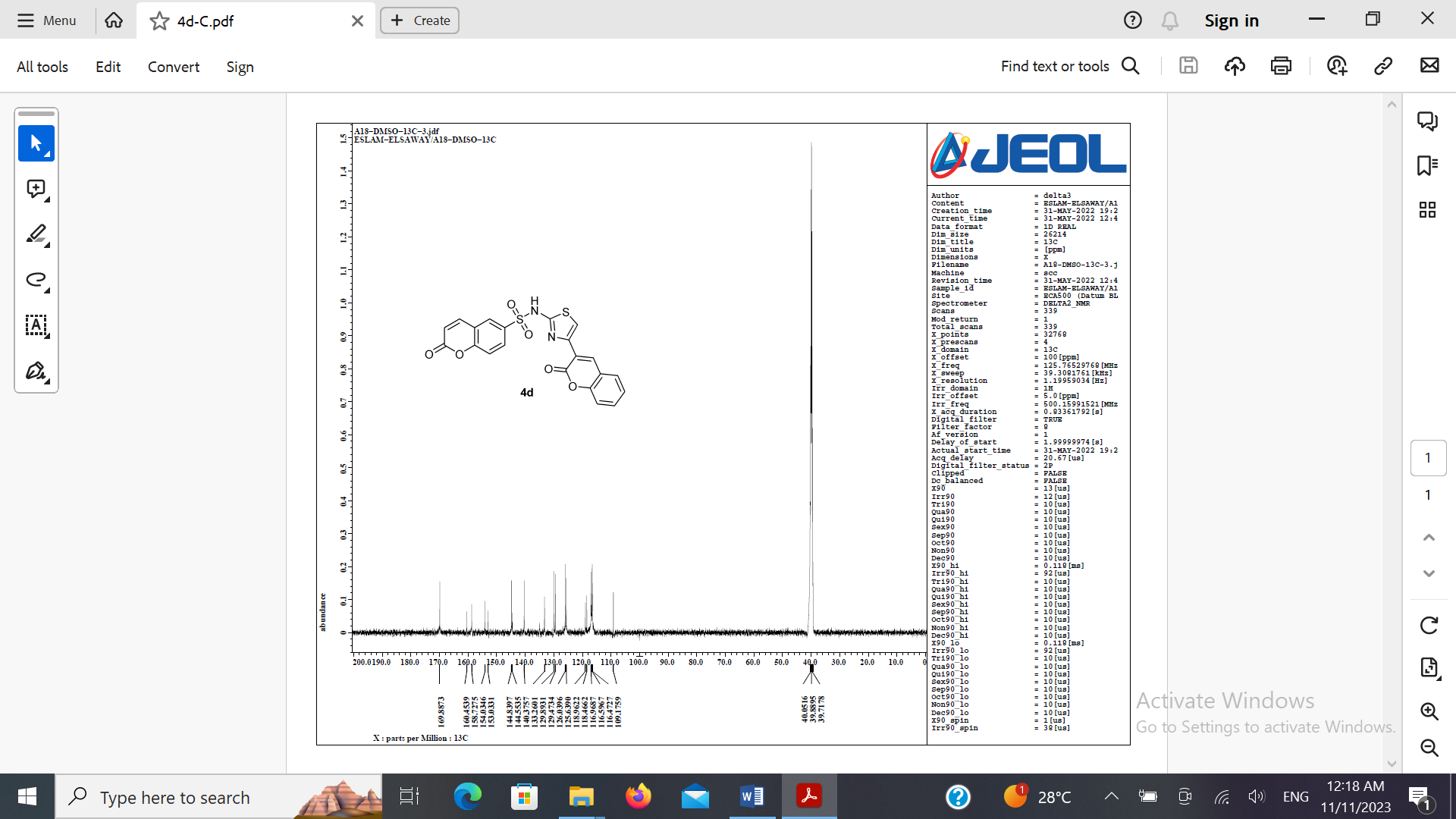


Figure s9. The ^13^C NMR (DMSO-d_6_) spectrum of compound **4d**


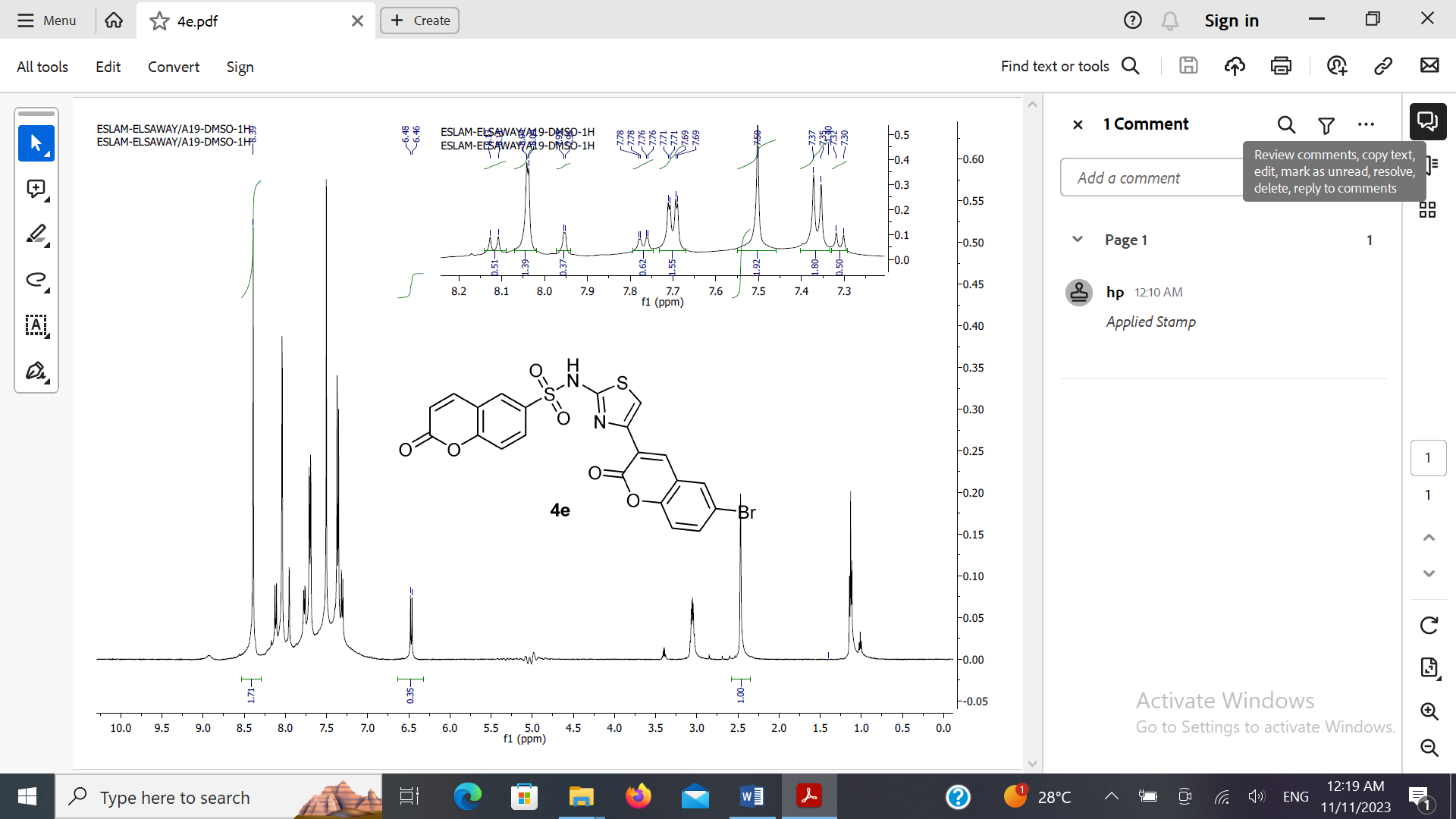


Figure s10. The ^1^H NMR (DMSO-d_6_) spectrum of compound **4e**


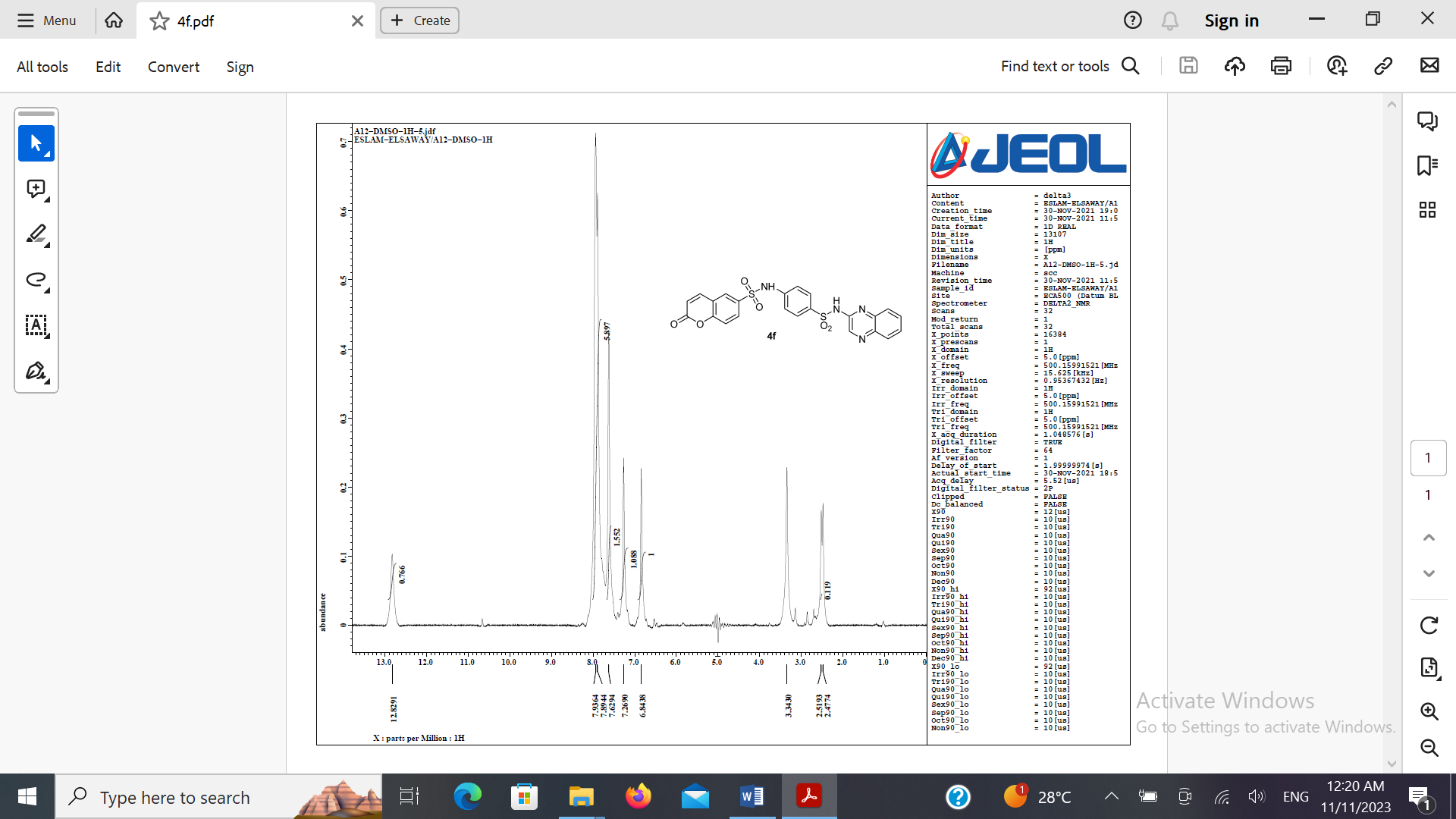


Figure s11. The ^1^H NMR (DMSO-d_6_) spectrum of compound **4f**


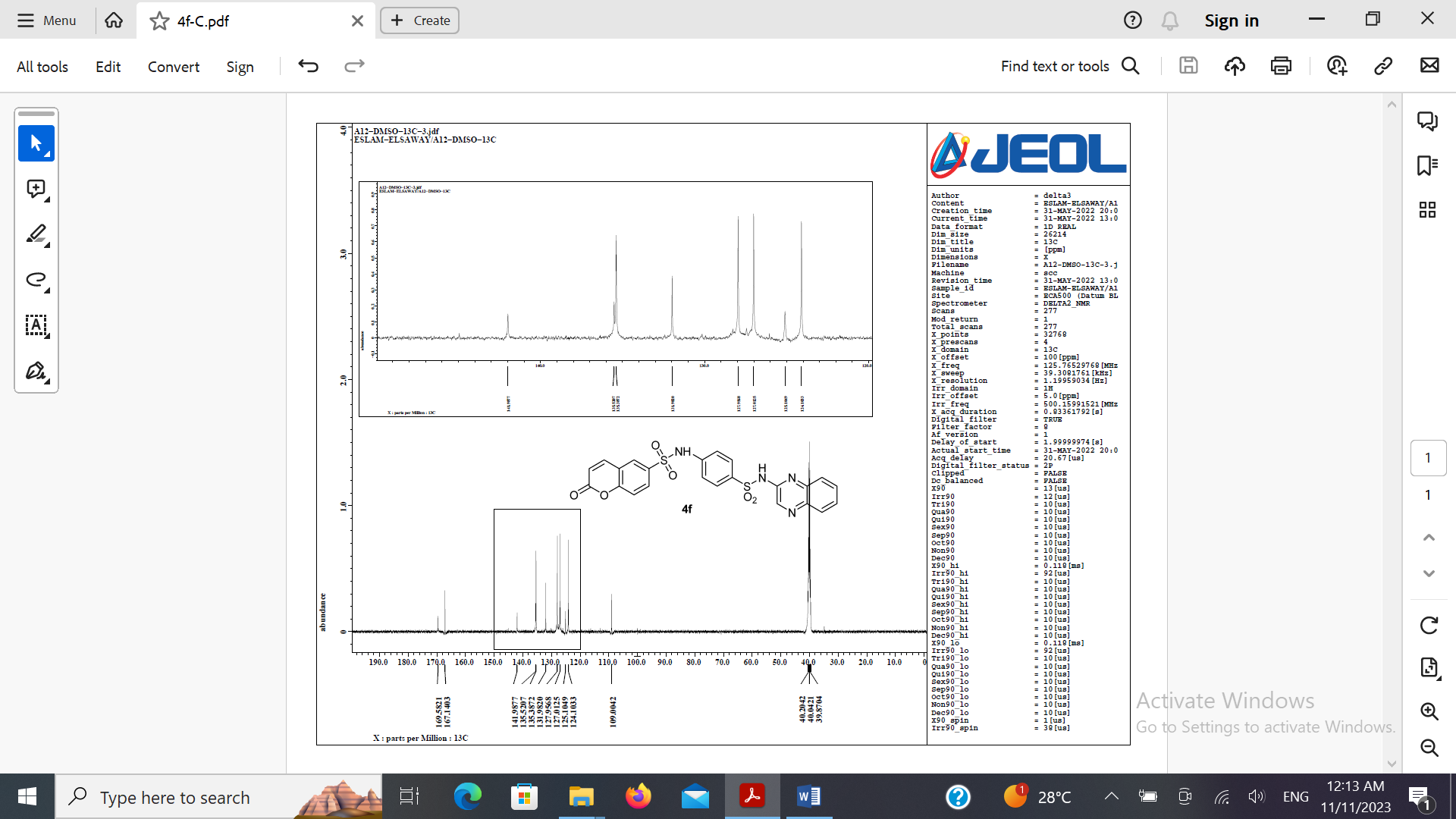


Figure s12. The ^13^C NMR (DMSO-d_6_) spectrum of compound **4f**


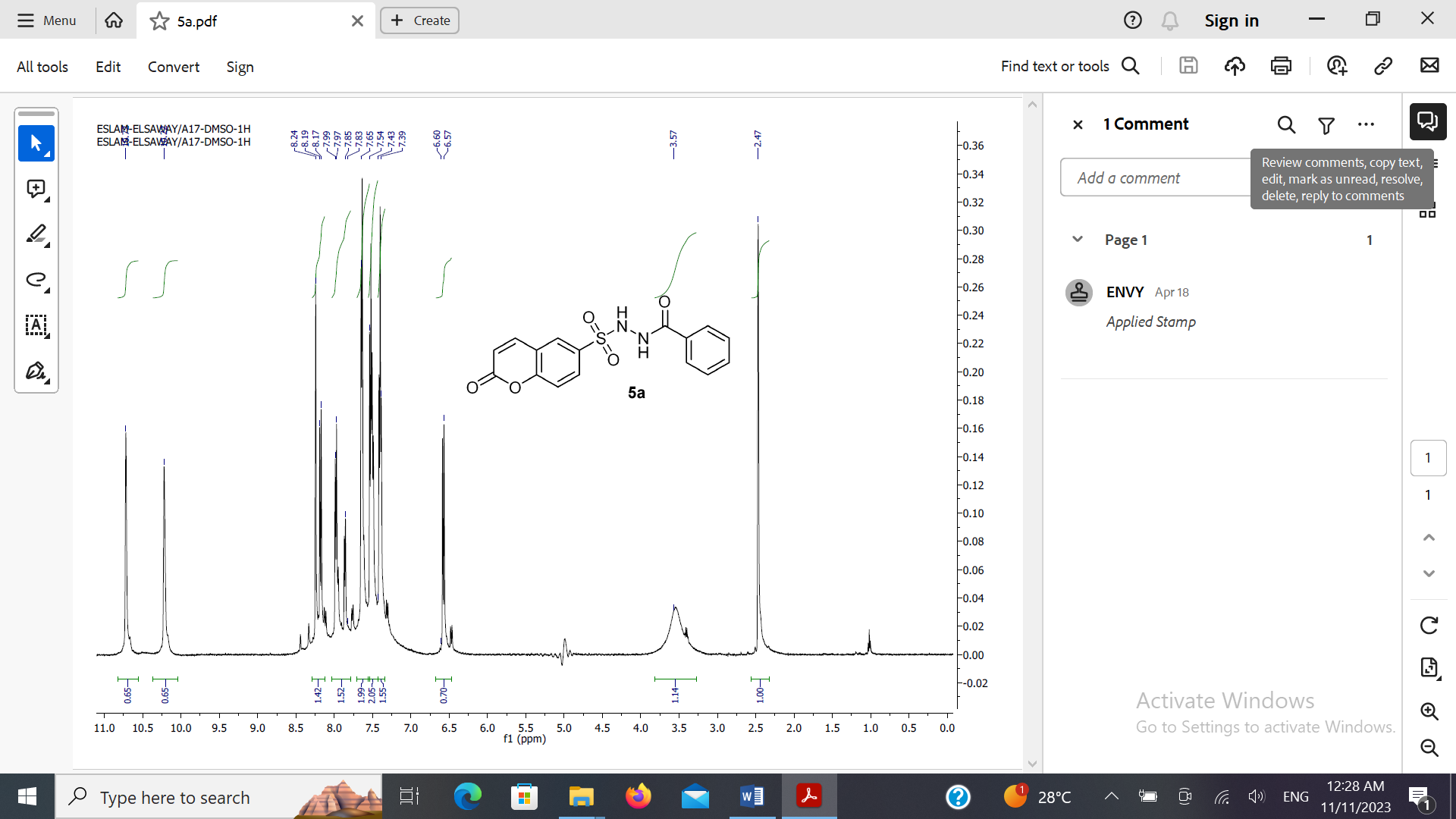


Figure s13. The ^1^H NMR (DMSO-d_6_) spectrum of compound **5a**


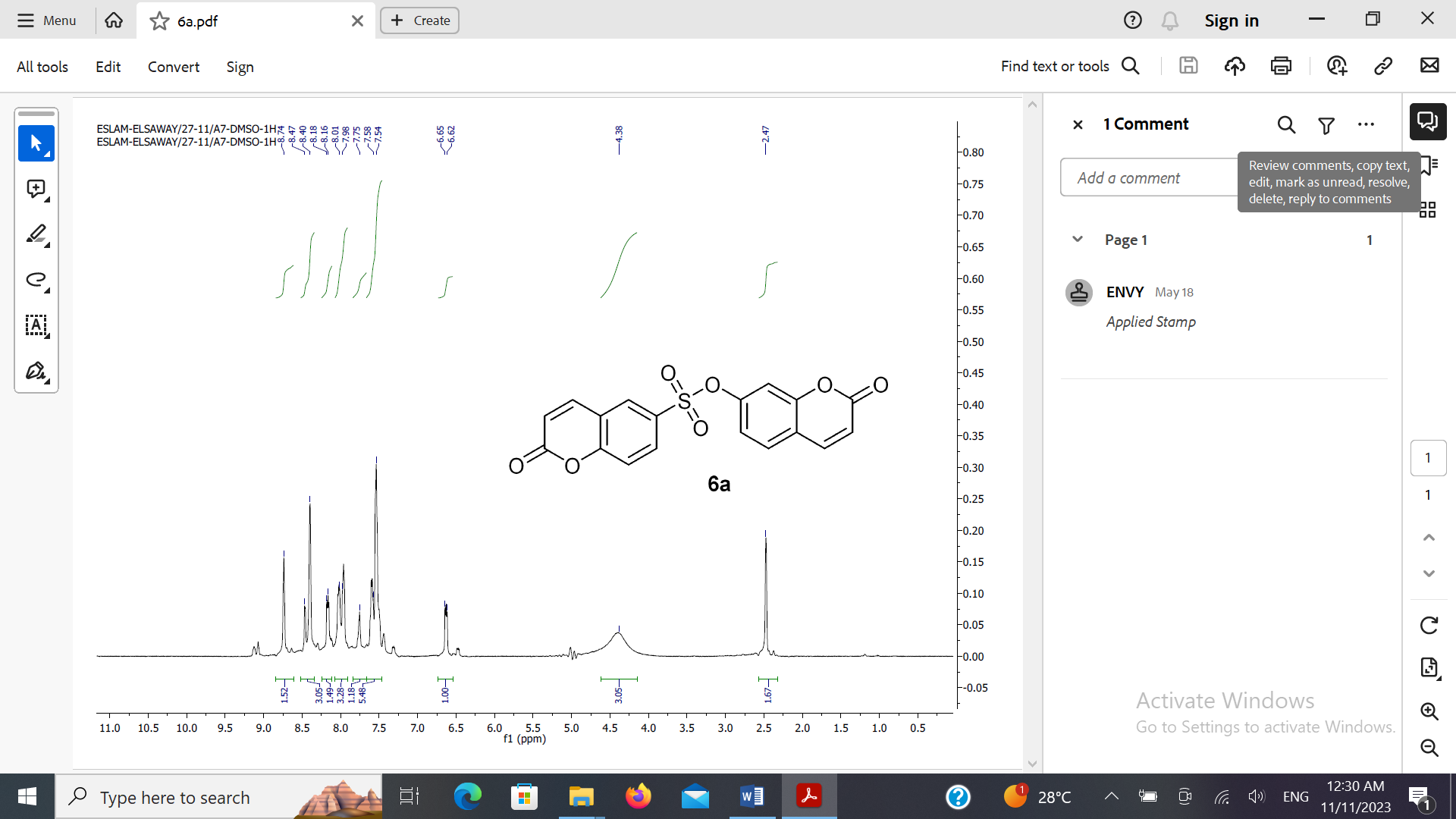


Figure s14. The ^1^H NMR (DMSO-d_6_) spectrum of compound **6a**


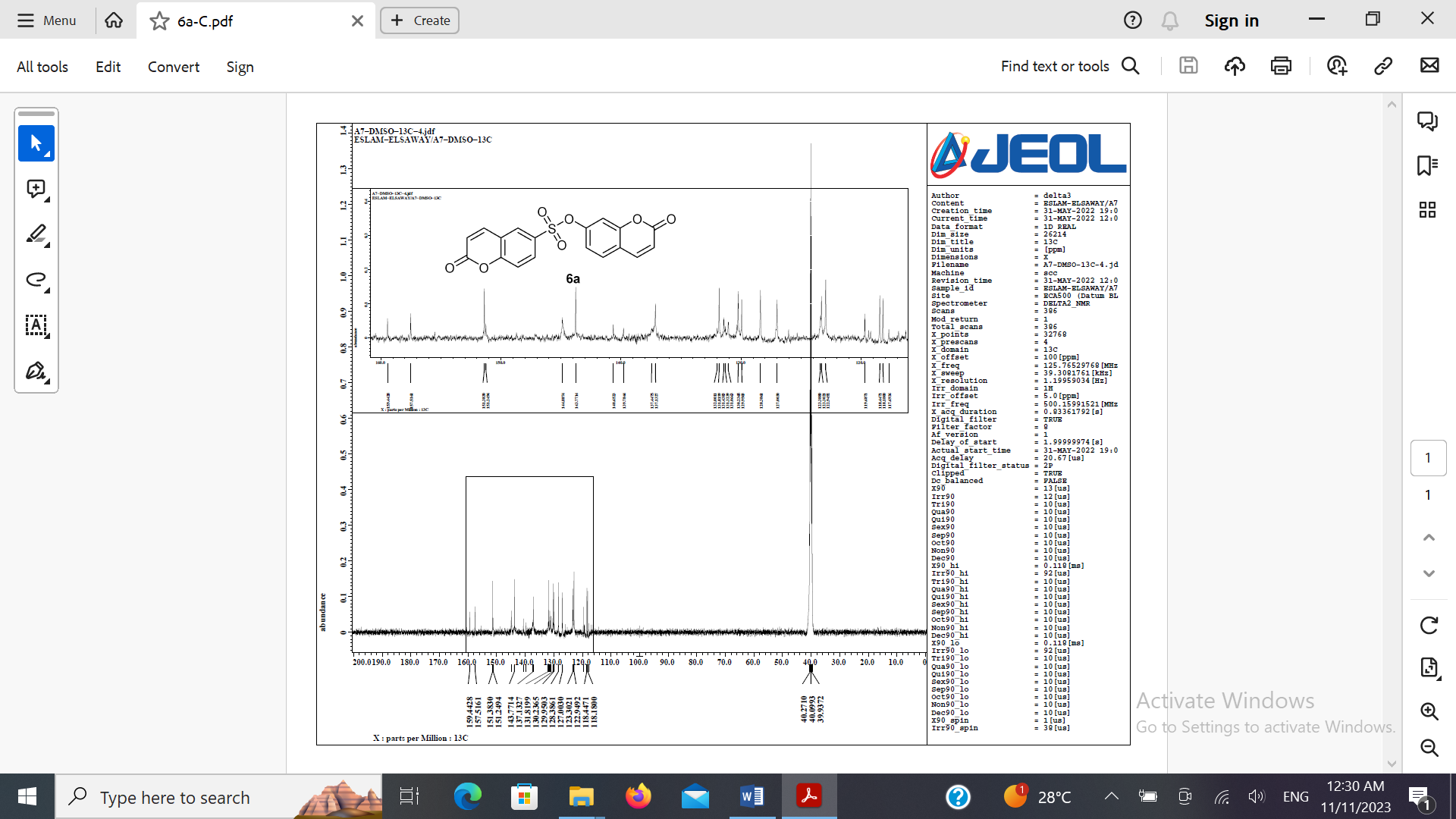


Figure s15. The ^13^C NMR (DMSO-d_6_) spectrum of compound **6a**


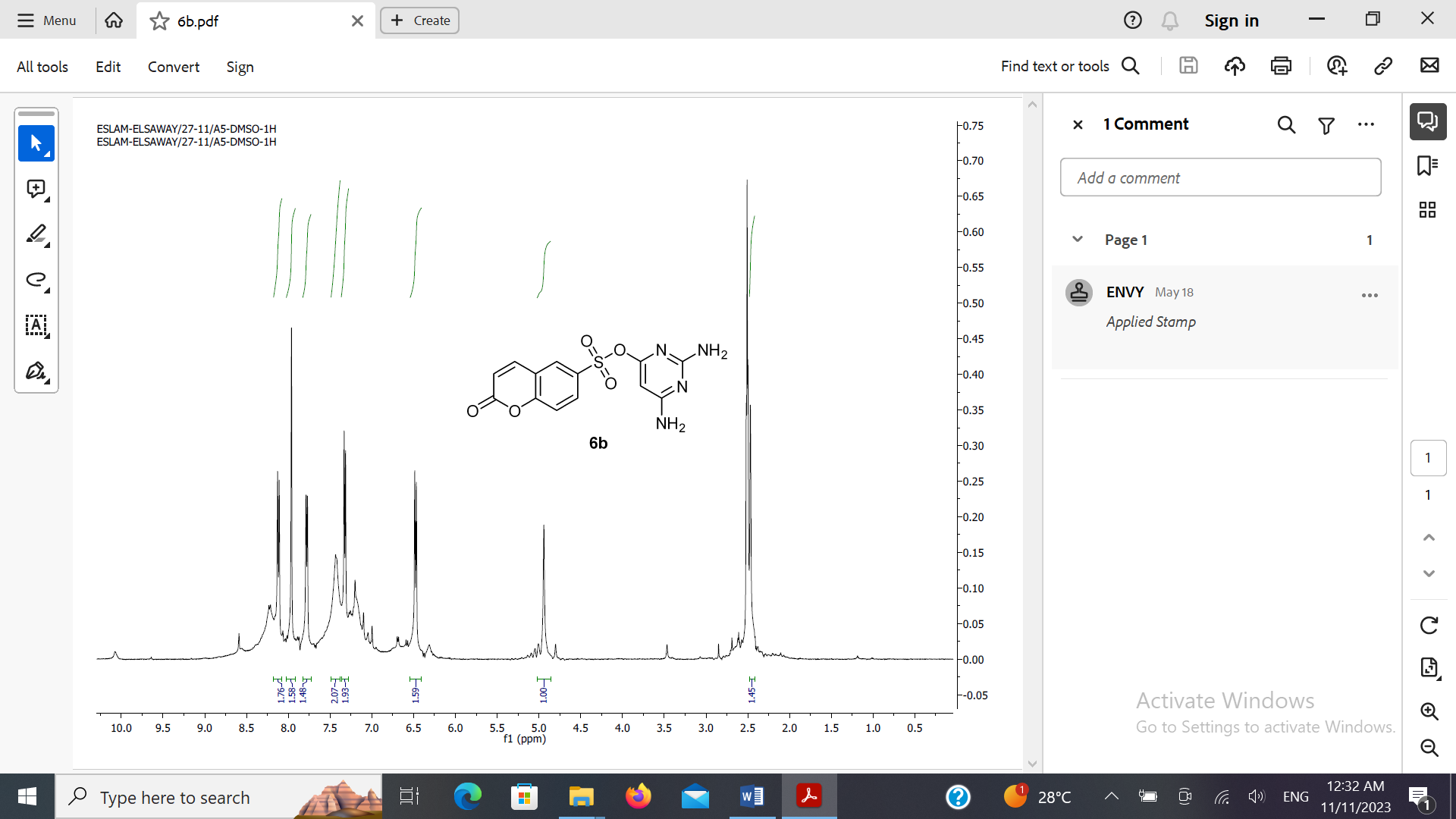


Figure s16. The ^1^H NMR (DMSO-d_6_) spectrum of compound **6b**


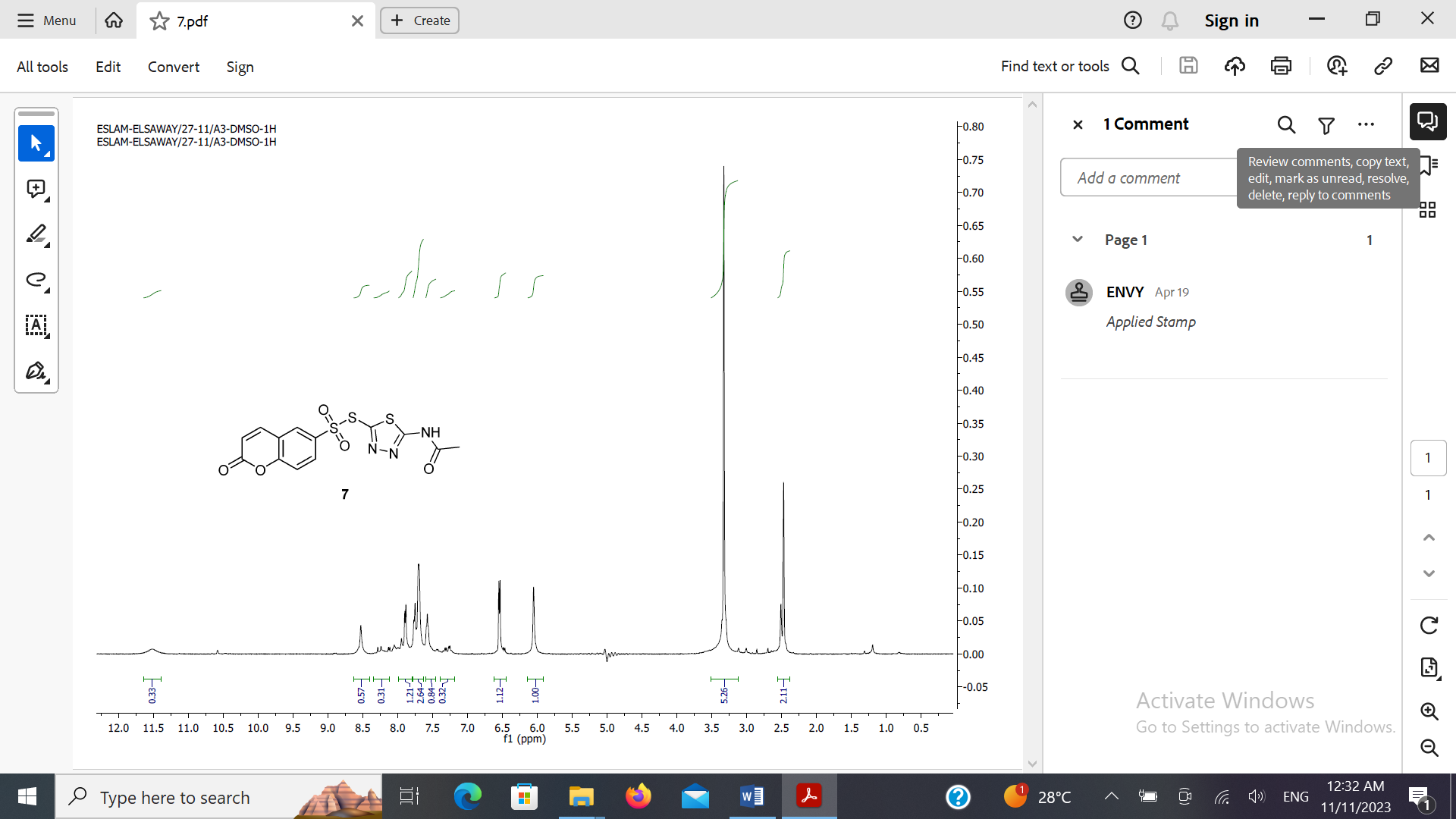


Figure s17. The ^1^H NMR (DMSO-d_6_) spectrum of compound **7a**


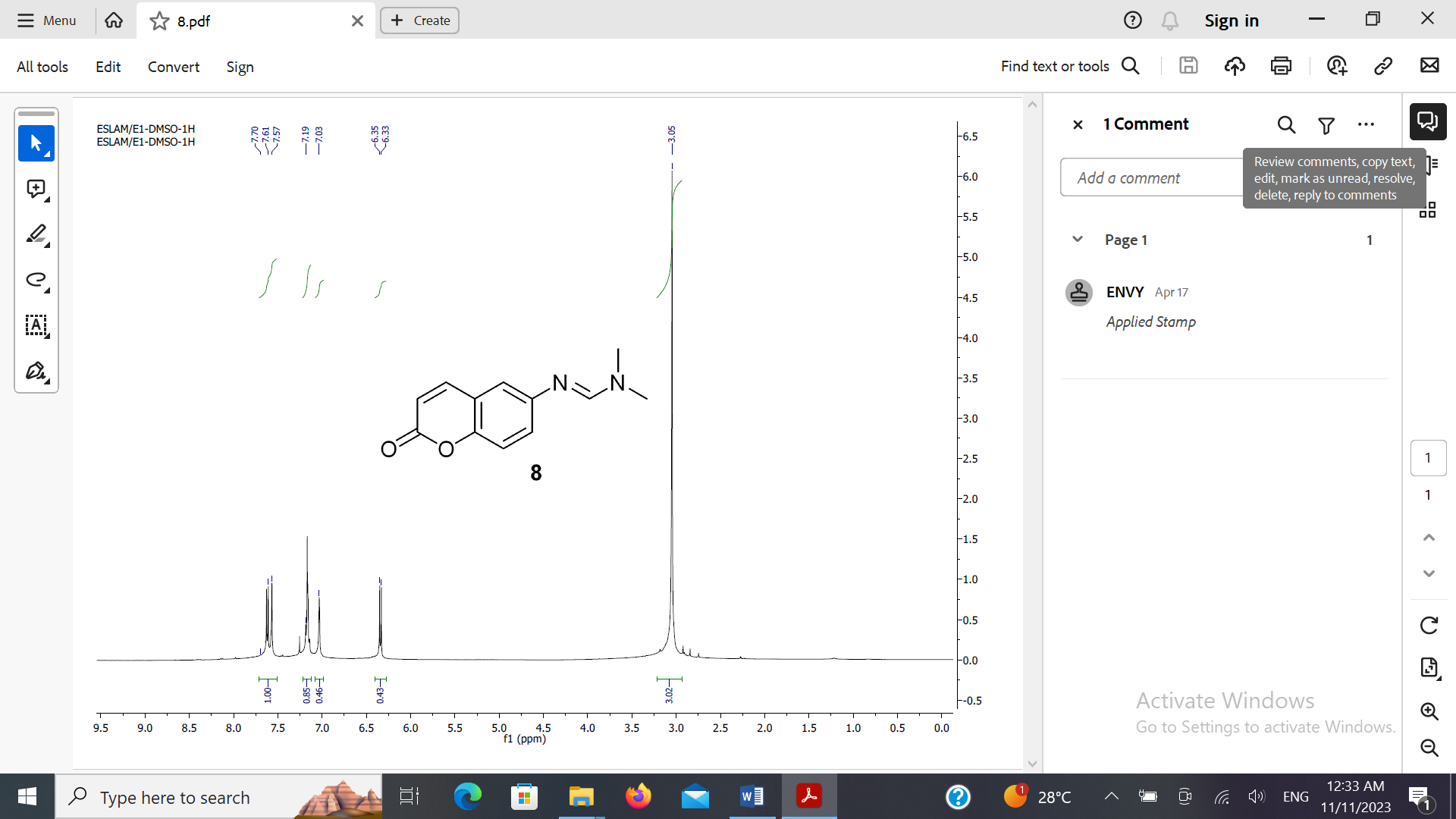


Figure s18. The ^1^H NMR (DMSO-d_6_) spectrum of compound **8**


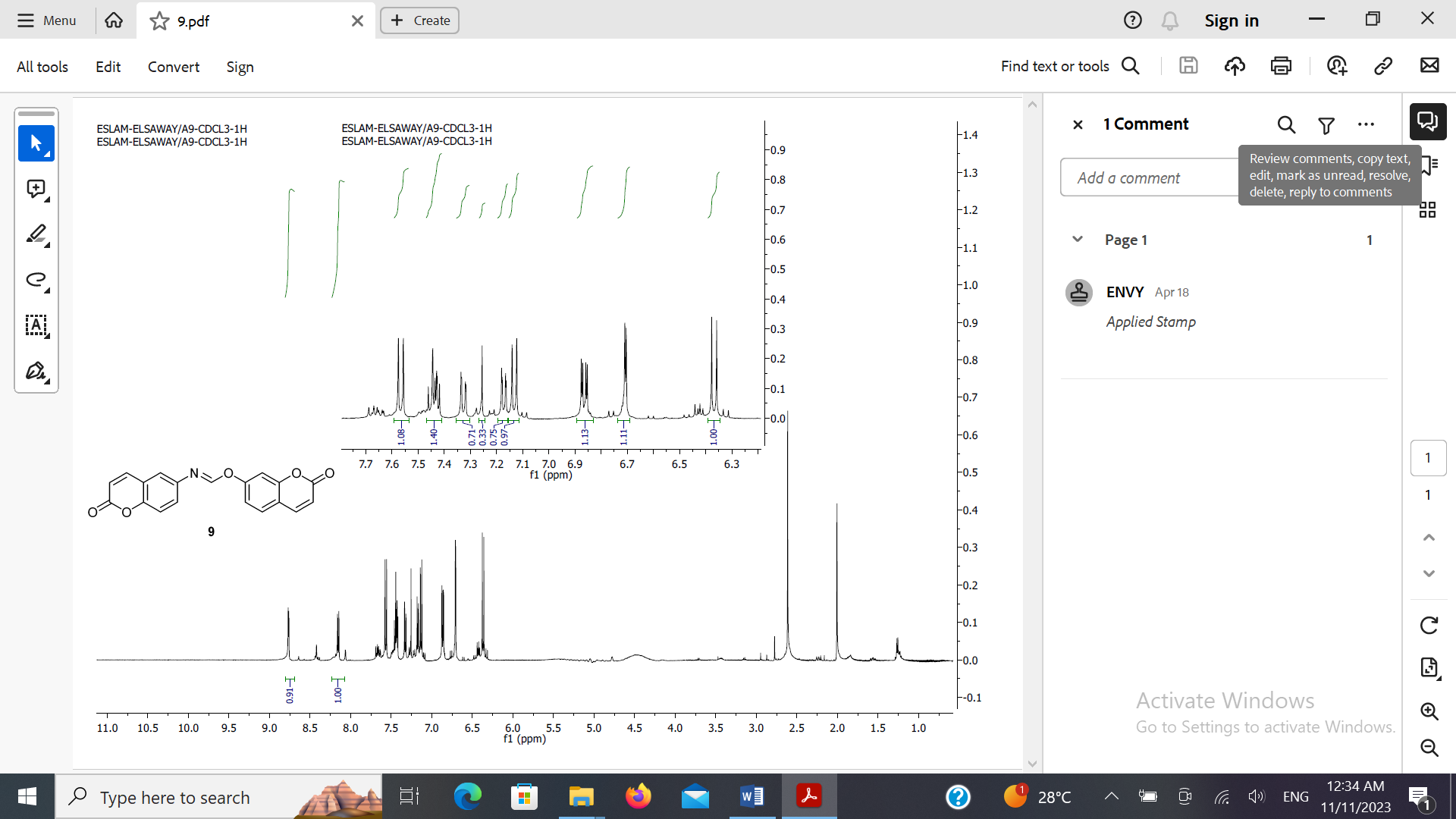


Figure s19. The ^1^H NMR (DMSO-d_6_) spectrum of compound **9**


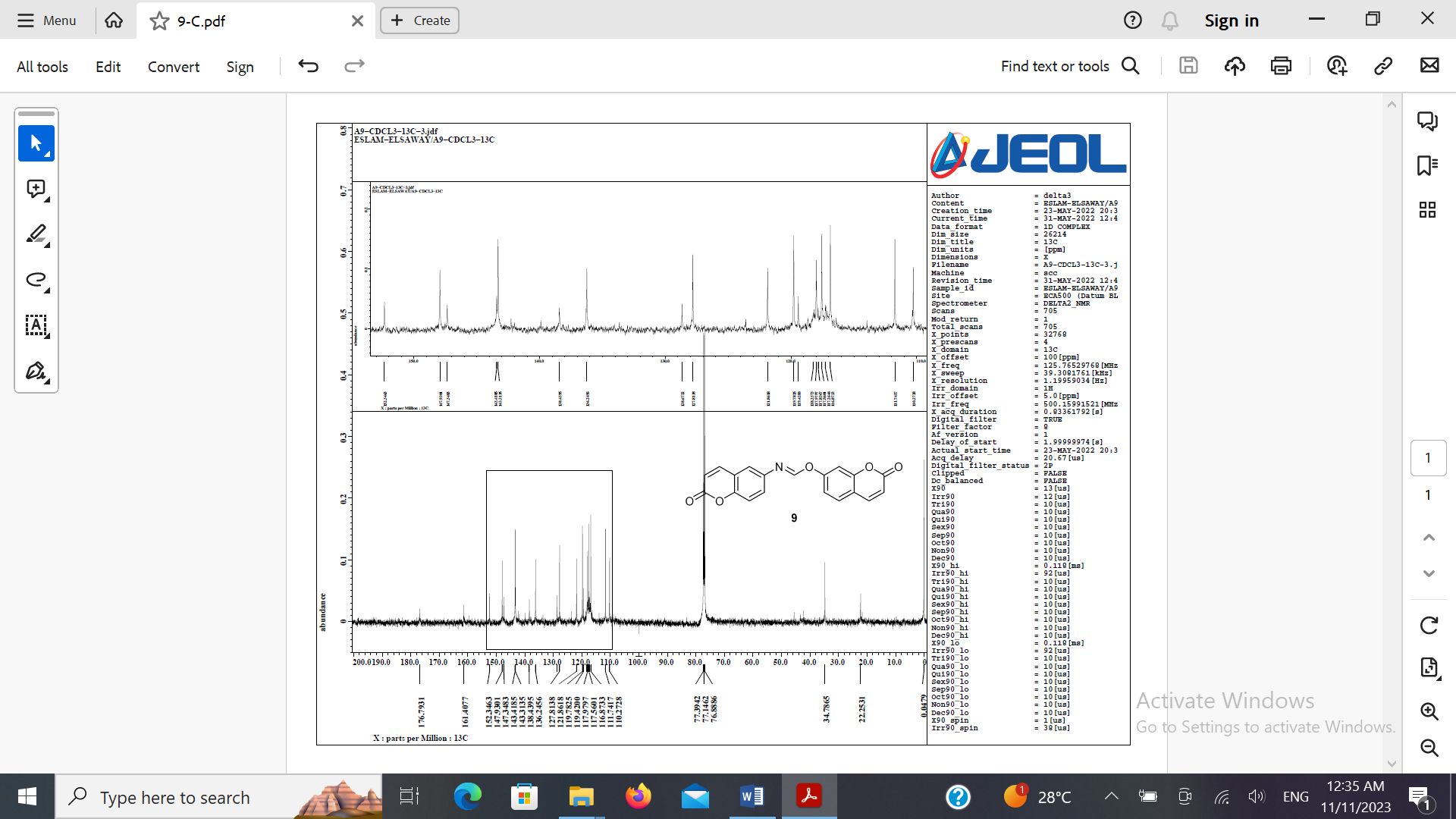


Figure s20. The ^13^C NMR (DMSO-d_6_) spectrum of compound **9**
